# Targeted Protein O-GlcNAcylation Using Bifunctional Small Molecules

**DOI:** 10.1021/jacs.3c14380

**Published:** 2024-04-01

**Authors:** Bowen Ma, Khadija Shahed Khan, Tongyang Xu, Josefina Xeque Amada, Zhihao Guo, Yunpeng Huang, Yu Yan, Henry Lam, Alfred Sze-Lok Cheng, Billy Wai-Lung Ng

**Affiliations:** † School of Pharmacy, Faculty of Medicine, The Chinese University of Hong Kong, Sha Tin, Hong Kong; ‡ School of Biomedical Sciences, Faculty of Medicine, The Chinese University of Hong Kong, Sha Tin, Hong Kong; § Li Ka Shing Institute of Health Sciences, Faculty of Medicine, The Chinese University of Hong Kong, Sha Tin, Hong Kong; ∥ Department of Chemical and Biological Engineering, The Hong Kong University of Science and Technology, Sai Kung, Hong Kong

## Abstract

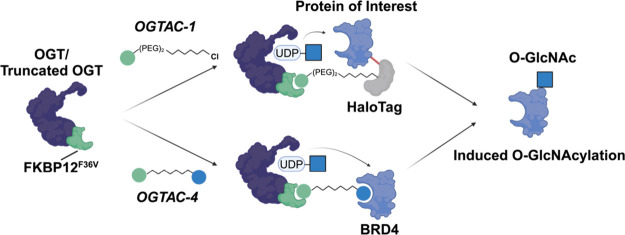

Protein O-linked β-*N*-acetylglucosamine modification (O-GlcNAcylation) plays a crucial role in regulating essential cellular processes. The disruption of the homeostasis of O-GlcNAcylation has been linked to various human diseases, including cancer, diabetes, and neurodegeneration. However, there are limited chemical tools for protein- and site-specific O-GlcNAc modification, rendering the precise study of the O-GlcNAcylation challenging. To address this, we have developed heterobifunctional small molecules, named O-GlcNAcylation TArgeting Chimeras (OGTACs), which enable protein-specific O-GlcNAcylation in living cells. OGTACs promote O-GlcNAcylation of proteins such as BRD4, CK2α, and EZH2 *in cellulo* by recruiting FKBP12^F36V^-fused O-GlcNAc transferase (OGT), with temporal, magnitude, and reversible control. Overall, the OGTACs represent a promising approach for inducing protein-specific O-GlcNAcylation, thus enabling functional dissection and offering new directions for O-GlcNAc-targeting therapeutic development.

## Introduction

Protein O-linked β-*N*-acetylglucosamine modification (O-GlcNAcylation) plays a significant role in the regulation of transcription, metabolism, cell signaling, protein stability, and nucleocytoplasmic trafficking.^1−4^ The abnormal regulation of global O-GlcNAcylation state is associated with many human diseases, including cancer,^4−8^ neurodegenerative diseases,^9^ cardiovascular diseases,^10^ autoimmune diseases,^11^ and diabetes.^12^

However, the functional roles of O-GlcNAcylation are protein-specific, and the dissection of the functional consequences of individual O-GlcNAc modification events remains highly challenging.^2,13^ Current strategies for studying protein-specific O-GlcNAcylation include glycosite genetic mutation,^14,15^ dual-specific RNA aptamers,^16^ and nanobody-induced OGT-substrate proximity *in cellulo*,^17,18^ which require extensive genetic engineering and are not amenable to magnitude control. Alternative techniques, such as protein semisynthesis by peptide–protein ligation, are useful in vitro but difficult to apply in a cellular setting.^19,20^

Chemical inhibitors of O-GlcNAc transferase (OGT) and O-GlcNAcase (OGA), the enzymes that catalyze the addition and removal of O-GlcNAc, have also been intensively studied.^21^ However, as OGT and OGA are the only pair of enzymes regulating the O-GlcNAcylation of thousands of nuclear and cytosolic proteins, the inhibition of these enzymes inevitably disrupts global cellular O-GlcNAcylation homeostasis, rendering the functional dissection and manipulation of protein-specific O-GlcNAcylation impossible. Moreover, prolonged treatment with these inhibitors can trigger feedback regulation of OGT/OGA, leading to confounding results from chemical perturbations.^22,23^ Inspired by the development of chemically induced protein post-translational modifications,^24−32^ we hypothesized that a heterobifunctional system that recruits OGT to the target protein could achieve target-specific O-GlcNAcylation. Unlike conventional OGA inhibitors, which directly block deglycosylation and thereby sustain high levels of O-GlcNAcylation, our heterobifunctional molecules are designed to bind the OGT and a protein of interest (POI) simultaneously, inducing their proximity and triggering POI-specific O-GlcNAcylation.

Here, we present the development of O-GlcNAcylation TArgeting chimeras (OGTACs), which selectively induce O-GlcNAcylation of (theoretically) any POI, as demonstrated here by BRD4, CK2α, and EZH2, through promoting proximity with the OGT. We optimize the molecular structure of the OGTACs and validate that their O-GlcNAcylation-inducing effect is dose- and time-dependent, reversible, and without obvious disruption of the global O-GlcNAc level. We envision that our OGTAC strategy will accelerate functional dissection and therapeutic development targeting protein O-GlcNAcylation.

## Results and Disscussion

### Design of General OGTACs to Induce Targeted Protein O-GlcNAcylation in Cells

**Figure 1:**
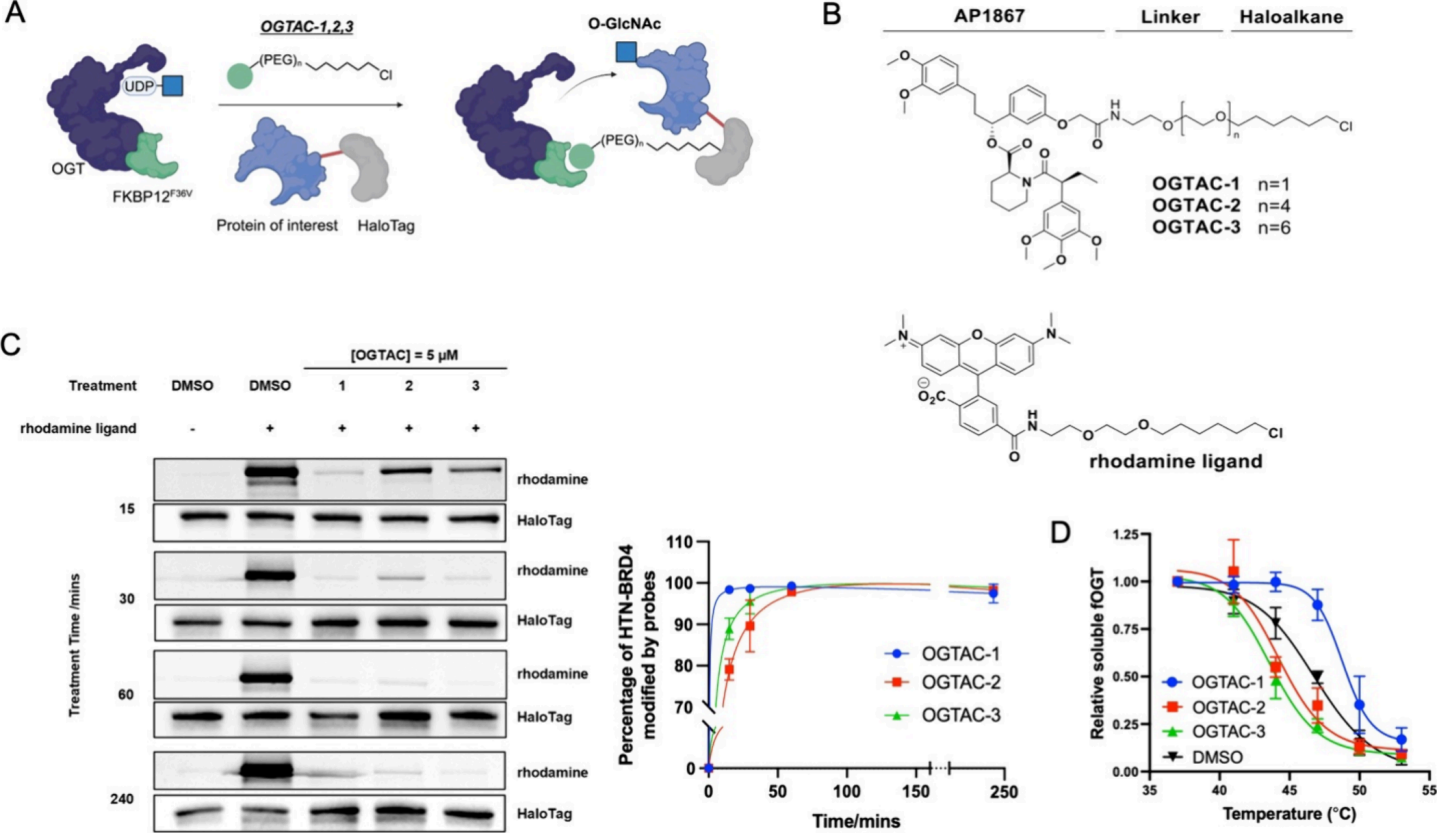
General concept of OGTAC technology and target engagement studies of OGTAC molecules. (A) Conceptual scheme of general OGTACs, which induce POI-specific O-GlcNAcylation by recruiting FKBP12^F36V^-OGT to HaloTag-fused POIs. (B) Chemical structure of OGTAC-1,2,3, and rhodamine ligand for the pulse-chase experiment. (C, D) Confirmation of OGTAC-1,2,3 target engagement in cells. HEK293T cells were transiently transfected with plasmids HTN-BRD4:fOGT at a 1:0.05 ratio. (C) Pulse-chase assay to verify the engagement of the OGTACs with HTN-BRD4. OGTAC-1,2,3 (5 μM) or vehicle was added to cells for increasing periods of time, followed by replacement with rhodamine ligand to label any remaining HTN-BRD4 proteins that were not engaged by OGTACs. Then, cells were lysed and subjected to SDS-PAGE for in-gel fluorescence using the rhodamine channel and immunoblotting using a HaloTag antibody to verify equal loading. (D) After 4 h treatment of 5 μM OGTAC-1,2,3, CETSA was conducted to verify their direct binding to fOGT. Data in (C, D) represent mean ± s.d. of *n* = 3 biologically independent replicates.

Unlike E3 ligases in ubiquitination or kinases in phosphorylation, which exhibit relatively high substrate specificity, OGT is a glycotransferase with broad substrate tolerance, modifying >5000 human cellular proteins.^33^ Therefore, we hypothesized that a general chemical-induced proximity strategy could be developed to induce O-GlcNAcylation of theoretically any POIs by harnessing the substrate promiscuity of OGT.^34,35^ To demonstrate the feasibility of inducing protein-specific O-GlcNAcylation, we first established a general tag-based system for a proof-of-concept study. Since OGT activity is required, and there are currently no noninhibitory ligands available for OGT, we exploited FKBP12^F36V^-fused OGT (fOGT) as the O-GlcNAc transferase,^36^ which can be efficiently recruited by the AP1867 motif. HaloTag-fused POIs were overexpressed as O-GlcNAcylation targets, which can be covalently captured by haloalkanes.^37^ We synthesized a series of molecules by connecting AP1867 and a haloalkane through various lengths of poly(ethylene glycol) (PEG) linkers, producing O-GlcNAcylation-TArgeting Chimeras (OGTACs) (Figure 1A,B). These OGTACs are designed to induce proximity between fOGT and any HaloTag-fused POIs while concurrently promoting O-GlcNAc transfer to these specific POIs.

### OGTAC-1 Induces BRD4 O-GlcNAcylation in a Dose- and Time-Dependent Manner

We selected bromodomain-containing protein 4 (BRD4) as the first POI for our proof-of-concept study. BRD4 is an epigenetic regulator that plays a significant role in cancer.^38^ Post-translational modifications (PTMs) of BRD4, such as phosphorylation, methylation, and ubiquitination, are well studied; its phosphorylation was reported to affect chromatin targeting, onco-factors recruitment, and cancer progression.^39^ However, the O-GlcNAcylation of BRD4 has never been studied comprehensively due to the lack of appropriate tools.

We first constructed the HaloTag-N-terminal BRD4 (HTN-BRD4) plasmid and cotransfected it with fOGT plasmid in HEK293T cells at different ratios ranging from 1:1 to 1:0.02. By immunoprecipitation (IP) and probing for O-GlcNAc using the RL2 antibody in Western blot (IP-WB), we observed that the O-GlcNAcylation level of HTN-BRD4 was proportional to the amount of fOGT expressed and commenced only when fOGT expression reached a threshold value (Figure S1). We, therefore, set the cotransfection ratio at 1:0.05, the threshold which showed minimal O-GlcNAcylation, for our OGTAC induction study.

Before the induction effects of the OGTACs were evaluated, two cell-based assays were established to verify their ability to engage HTN-BRD4 and fOGT in cells. As the OGTAC-1/2/3 were designed to be irreversibly linked to the HaloTag, we developed a pulse-chase assay to assess their HTN-BRD4 engagement efficacy. We first treated HEK293T cells coexpressing HTN-BRD4 and fOGT with OGTACs for various durations; then, we replaced the culture media and exposed the cells to a rhodamine ligand, which fluorescently labeled the HTN-BRD4 that did not interact with OGTACs (Figure 1C). Four time points were selected for the pulse of the OGTAC treatment, and the rhodamine signal on HTN-BRD4 was evaluated by in-gel fluorescence; a weaker rhodamine signal indicated that more of the OGTACs had already reacted with the HaloTag, thereby blocking the binding of the rhodamine ligand. All three OGTACs efficiently engaged HTN-BRD4, achieving near-complete labeling of the protein within 4 h. OGTAC-1 was the most efficient among the three OGTACs, achieving >90% labeling within 15 min of treatment.

**Figure 2:**
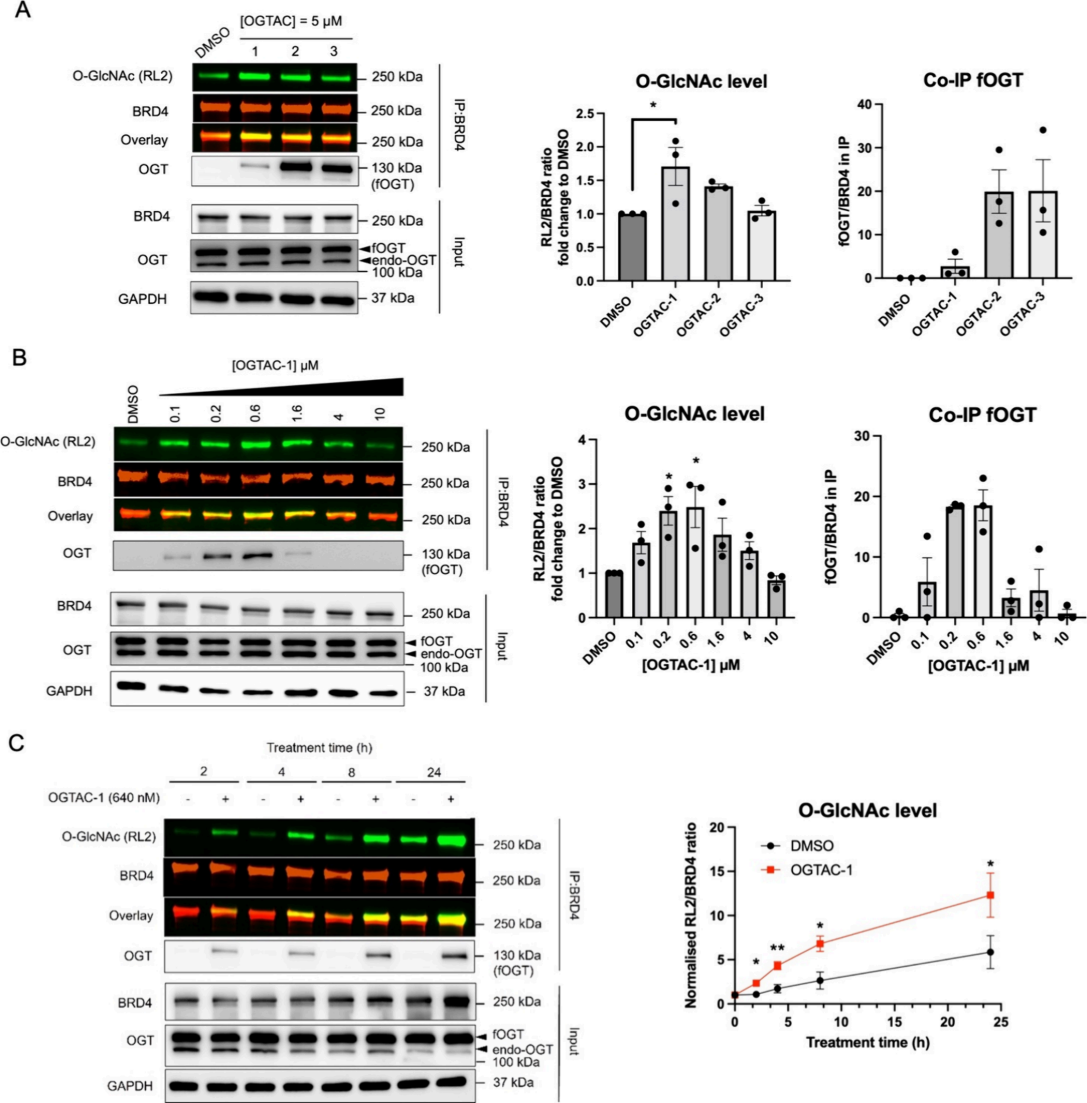
OGTAC-1 induces dose-dependent, rapid, targeted O-GlcNAcylation of HTN-BRD4 by recruiting fOGT in cells. (A) OGTAC-1 showed the best O-GlcNAcylation-inducing potency among three OGTACs at 5 μM. Endo-OGT, endogenous OGT. (B) Dose-dependent O-GlcNAcylation profile of HTN-BRD4 by the OGTAC-1 treatment. HEK293T cells expressing HTN-BRD4:fOGT = 1:0.05 were treated with increasing concentration of OGTAC-1 for 4 h. Then, the O-GlcNAcylation level of HTN-BRD4 was assessed by immunoblot after IP using BRD4 antibody. Co-IP of fOGT was also observed in an OGTAC-1 dose-dependent manner; (C) OGTAC-1 (640 nM) O-GlcNAcylation-inducing effects on HTN-BRD4 over the indicated time course. Notably, the marginal divergence between the red and black curves after 4 h suggests a diminished effect of OGTAC-1. For (A) and (B), the right panels show the quantifications of the immunoblot signal of RL2 relative to HTN-BRD4 and Co-IPed fOGT relative to IPed BRD4, as the mean ± s.e.m. of *n* = 3 biologically independent experiments. Statistical significance of (A) and (B) was calculated with ordinary one-way ANOVA; that of (C) was calculated with unpaired multiple *t* test. *p < 0.05; **p < 0.01.

To assess fOGT engagement, we established a cellular thermal shift assay (CETSA). Using the same coexpression system, we treated cells with OGTAC-1/2/3 for 4 h, and the intact cells were subjected to a heat challenge at various temperatures to determine the corresponding melting temperature (*T*_m_). The groups treated with the OGTAC-1 showed increased *T*_m_ compared with the DMSO control, with Δ*T*_m_ = 2 °C (Figures 1D andS2), indicating that the OGTAC-1 bound to the fOGT protein and thereby stabilized the protein. However, OGTAC-2/3 showed no stabilizing effect on fOGT. Since CETSA measures the relative amount of soluble protein after heat shock, we suspect that OGTAC-2 and 3 did not increase *T*_m_ due to the stable ternary complex they induced (shown in the next section, Figure 2A). We suspect that the large ternary complex (molecular weight ∼ 400 kDa) is more likely to become insoluble upon heat challenge above 41 °C.

We next assessed the induction of the O-GlcNAcylation of HTN-BRD4 in cells. We treated cotransfected HEK293T cells with OGTAC-1/2/3 for 4 h, followed by immunoprecipitation of BRD4 proteins from the cell lysate and measurement of the O-GlcNAcylation level by immunoblot with RL2 antibody. All three OGTACs induced O-GlcNAcylation on HTN-BRD4, with OGTAC-1 showing the highest potency (Figure 2A), consistent with the pulse-chase assay and CETSA.

We further investigated the dose-dependent effect of the OGTAC-1. The inducing effect exhibited a bell-shaped curve, with the highest level of O-GlcNAcylation observed between 256 and 640 nM (Figure 2B). Co-IP of fOGT was also observed at these concentrations, indicating that the induction of HTN-BRD4 in the O-GlcNAcylation depends on stable ternary complex formation (Figure 2B). The O-GlcNAcylation-inducing effect of OGTAC-1 declined from 1.6 μM, demonstrating that a hook effect occurred at this concentration.

Following the identification of the optimized concentration of OGTAC-1, we evaluated the kinetics of the OGTAC-mediated O-GlcNAcylation to investigate time-dependent effects. We examined the O-GlcNAcylation level at 2, 4, 8, and 24 h. The RL2 signal of HTN-BRD4 displayed a 2.5-fold increase at 2 h and further escalated to a 3-fold increase at 4 h (Figure 2C). However, the effect of OGTAC-1 gradually diminished over longer treatment durations, possibly due to the limitation of the transient transfection system: although media with transfection reagents and plasmids were removed before OGTAC treatment, the cells continued to express exogenous HTN-BRD4 and fOGT. As a result, the optimized ratio of fOGT:OGTAC:HTN-BRD4 for 4 h of treatment was compromised, leading to weaker potency of the inducing effect for longer treatment durations. Collectively, these data suggest that the OGTAC-1 induced BRD4 O-GlcNAcylation in a dose-dependent and time-dependent manner.

### OGTAC Strategy Generally Applied to Other POIs

**Figure 3:**
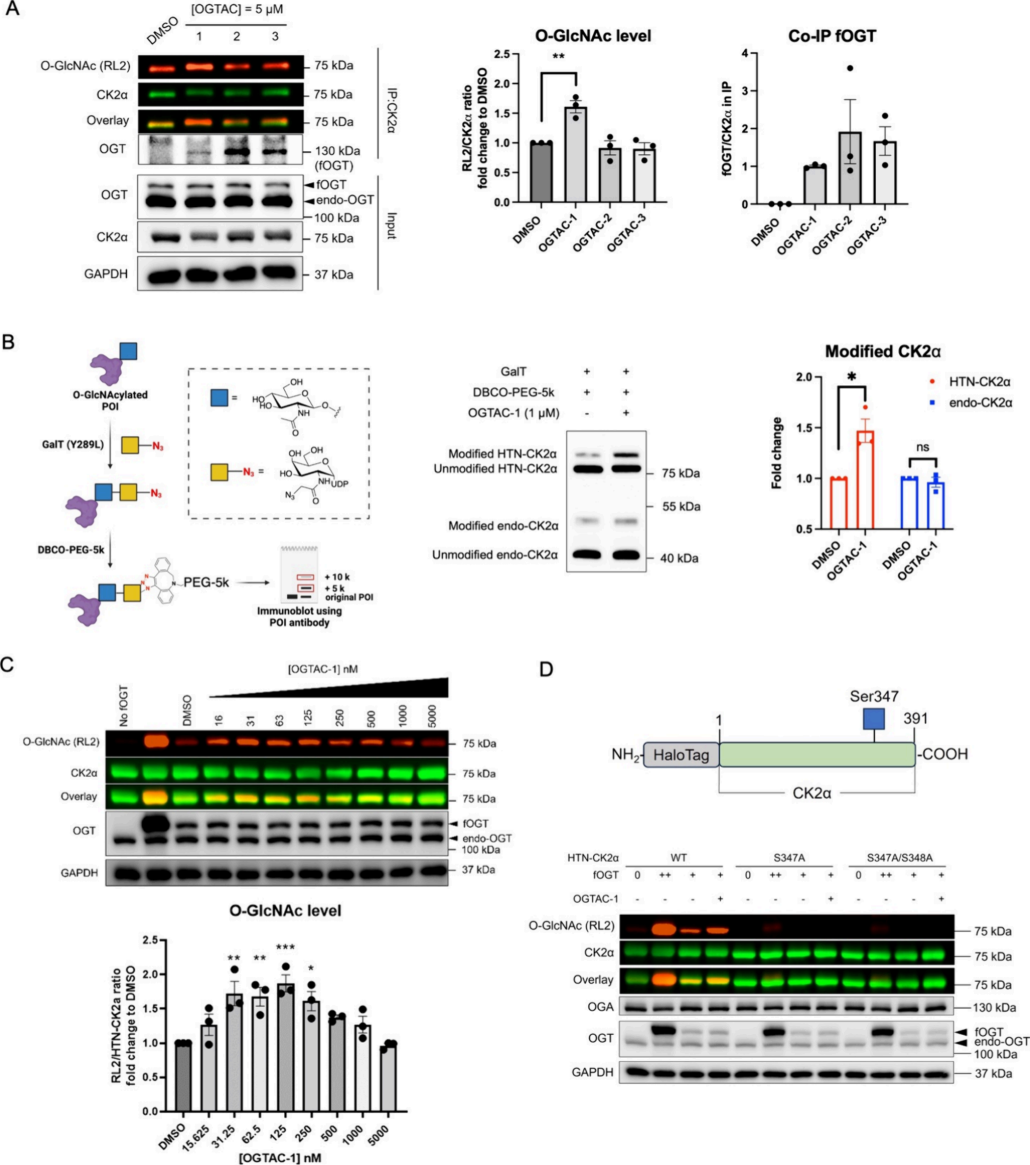
OGTAC technology extended to HTN-CK2α. (A) IP-WB method showed OGTAC-induced HTN-CK2α O-GlcNAcylation. (B) Mass shift assay to validate the OGTAC-1-induced specific HTN-CK2α O-GlcNAcylation (data for other two repeats can be found in Figure S4); (C) OGTAC-1-induced dose-dependent O-GlcNAcylation of HTN-CK2α in cells. HEK293T cells were transfected with HTN-CK2α:fOGT = 1:0.01 for 24 h, followed by treatment with increasing concentration of OGTAC-1 for 8 h. Transfection with HTN-CK2α:fOGT = 1:0.2 was set as positive control; (D) OGTAC-1-induced physiologically relevant S347 O-GlcNAcylation in HTN-CK2α. “++” denotes the HTN-CK2α:fOGT = 1:0.2, while “+” denotes the HTN-CK2α:fOGT = 1:0.01. All quantifications are shown as mean ± s.e.m. of 3 independent biological repeats. Statistical significance for (B) was calculated with two-tailed Student’s *t* test, and remaining significance were calculated with ordinary one-way ANOVA comparing DMSO- to OGTAC-1-treated samples. ns, *p* ≥ 0.05; *p < 0.05; **p < 0.01; ***p < 0.001.

Next, to illustrate the generality of our OGTAC strategy, we assessed the effects of OGTAC-1/2/3 on HTN-Casein kinase alpha (HTN-CK2α). CK2α is a well-documented substrate of OGT, and O-GlcNAcylation on its Ser347 residue was reported to modulate its kinase substrate selectivity.^18,19,40^ We first coexpressed HTN-CK2α and fOGT in HEK293T cells at various ratios and found that the basal O-GlcNAcylation level of HTN-CK2α was higher than that of HTN-BRD4. Therefore, a lower ratio of fOGT expression (1:0.01) was selected to assess OGTAC potency (Figure S3). We first conducted IP-WB, and the result indicated that OGTAC-1 was again the most efficient among the three OGTACs, despite yielding the lowest co-IP of fOGT (Figure 3A). The induction of specific O-GlcNAcylation on HTN-CK2α was further confirmed by a mass shift assay. In this assay, O-GlcNAcylated proteins in cell lysate were enzymatically labeled with N-azidoacetylgalactosamine (GalNAz), followed by covalent linkage to a 5 kDa dibenzocyclooctyne (DBCO)-PEG chain through strain-promoted alkyne-azide cycloaddition (SPAAC), resulting in an up-shifted band in immunoblot (Figures 3B and S4).^41^ We validated that 1 μM of OGTAC-1 increased HTN-CK2α O-GlcNAcylation; importantly, OGTAC-1 treatment did not alter the O-GlcNAcylation level of endogenous CK2α, suggesting its target specificity (Figures 3B and S4).

During the immunoblotting study, we noticed a quantifiable O-GlcNAcylation level (as indicated by RL2 signal) on HTN-CK2α in whole cell lysate, corresponding with IP-WB results (Figures S5, S6A and 3A). Therefore, in subsequent studies, we directly quantified the RL2 signal intensity on HTN-CK2α without IP. To validate the importance of direct binding to both fOGT and HTN-CK2α, we exploited competitors containing an AP1867 moiety and a rhodamine moiety, which bind to fOGT and HTN-CK2α, respectively, to compete with the sulfate peptide in OGTAC-1. As expected, cotreatment with either competitor abolished the O-GlcNAcylation-inducing effects of OGTAC-1 (Figure S6B).

Next, we investigated the dose- and time-dependent effects of the OGTAC-1 on HTN-CK2α. We observed that, at 125 nM, induction of O-GlcNAcylation occurred within 4 h, peaked at 8 h, and then started to decline (Figures 3C and S7–S9). However, for the 24 h treatment, the induction peaked at a higher concentration of OGTAC-1 (1 μM) instead (Figure S8). This might be due to the need for a larger population of OGTAC-1 molecules to maintain the appropriate stoichiometry with the increased expression of HTN-CK2α protein. This phenomenon again highlights the importance of optimizing the fOGT:OGTAC:POI ratios for efficient chemically induced O-GlcNAcylation.

S347 is a well-defined O-GlcNAcylation site on CK2α, while S348 is the potential alternative site as it is adjacent to S347.^18,19^ To verify the O-GlcNAc sites induced by OGTAC-1 in HTN-CK2α, we constructed HTN-CK2α plasmids with S347A and S347A/S348A mutations. Immunoblotting with the RL2 antibody indicates that S347 is a potential O-GlcNAc site on HTN-CK2α, with OGTAC-1 inducing O-GlcNAcylation at this site (Figure 3D). However, we must acknowledge the possibility of additional O-GlcNAc sites, given the known motif preferences and biases of RL2 antibody.^42,43^ Furthermore, the application of the OGTAC strategy in HeLa cells has also confirmed the effect of OGTAC-1 on HTN-CK2α (Figure S10), thereby ruling out the possibility that its action is limited to a single cell line. Collectively, these findings support the physiological relevance of the induced O-GlcNAcylation by OGTAC-1.

The same strategy was also extended to the enhancer of zeste homologue 2 (EZH2), an epigenetic regulator that is an OGT substrate.^44,45^ OGTAC-1 also demonstrated the highest O-GlcNAcylation-inducing effects among the three OGTACs (Figure S11). As there are >20,000 commercially available plasmids for HaloTag-fused human proteins,^46^ OGTACs can be rapidly tested in a wide range of POIs, enabling time- and dose-dependent manipulation of their specific O-GlcNAcylation level.

### OGTAC-4 Induces BRD4-Specific O-GlcNAcylation

**Figure 4:**
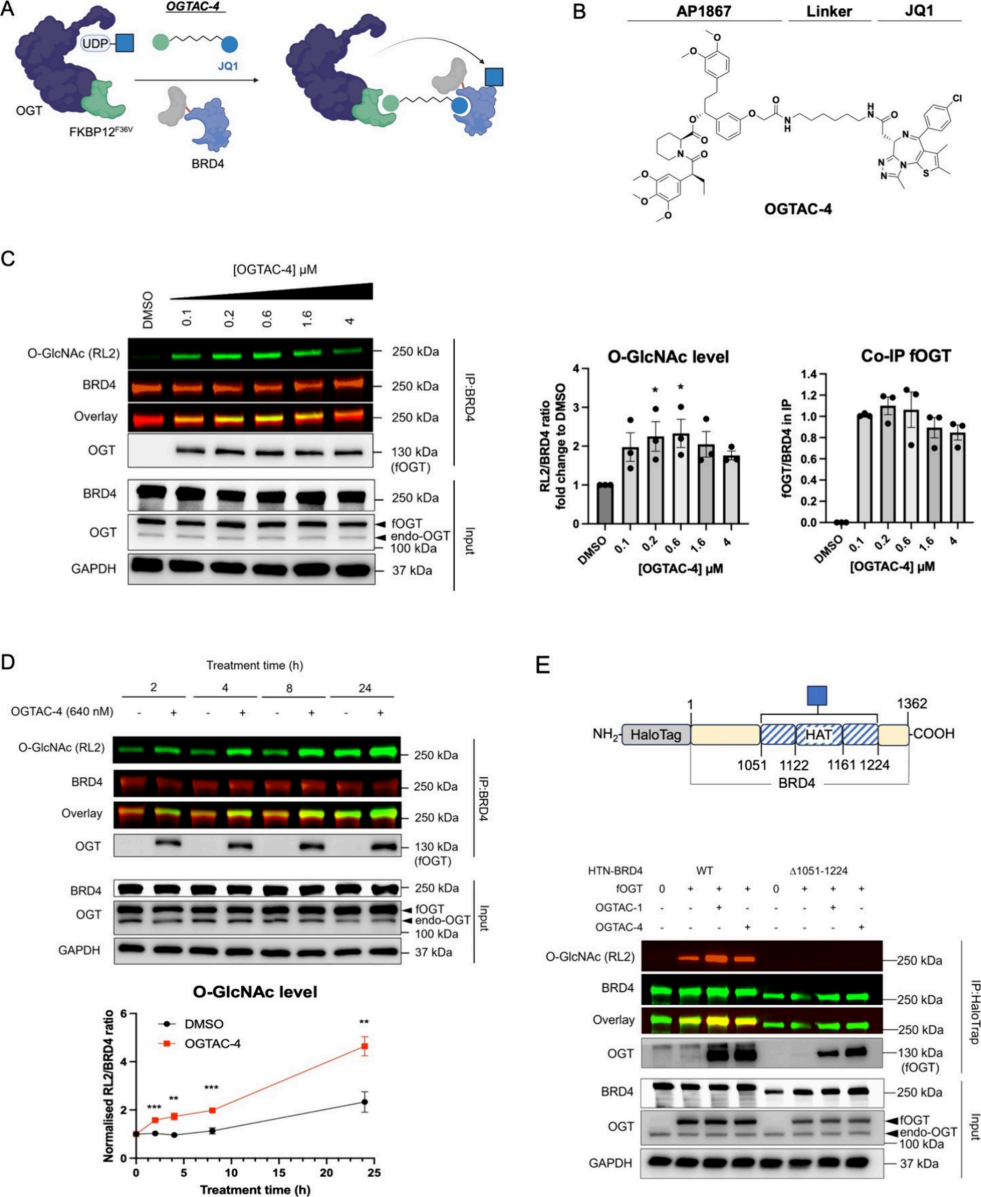
OGTAC-4 induces BRD4 O-GlcNAcylation by recruiting fOGT in cells. (A) Conceptual scheme of OGTAC-4, which induces BRD4-specific O-GlcNAcylation by recruiting FKBP12^F36V^-OGT to BRD4 through the JQ1 motif. (B) Chemical structure of OGTAC-4. (C) Dose-dependent O-GlcNAcylation profile of HTN-BRD4 by OGTAC-4 treatment. HEK293T cells expressing HTN-BRD4:fOGT = 1:0.05 were treated with an increasing concentration of OGTAC-4 for 4 h. Then, the O-GlcNAcylation level of HTN-BRD4 was assessed by immunoblot after IP using BRD4 antibody. Co-IP of fOGT was also observed in an OGTAC-4 dose-dependent manner; (D) OGTAC-4 (640 nM) O-GlcNAcylation-inducing effects on HTN-BRD4 over the indicated time course; (E) OGTAC-1 and OGTAC-4 induced HTN-BRD4 O-GlcNAcylation in the C-terminal region (S1367-F1224). “+” denotes the HTN-BRD4:fOGT = 1:0.05. The rectangles with blue stripes represent the truncated fragment used for validating O-GlcNAcylation sites. HAT, histone acetyltransferase catalytic domain; statistical significance was calculated with ordinary one-way ANOVA comparing DMSO- to OGTAC-4-treated samples. The quantifications of the immunoblot show the signal of RL2 relative to HTN-BRD4 and Co-IPed fOGT relative to IPed BRD4, as the mean ± s.e.m. of *n* = 3 biologically independent experiments. Statistical significance for (C) was calculated with ordinary one-way ANOVA; for (D), it was calculated with unpaired multiple *t* test. *p < 0.05; **p < 0.01; ***p < 0.001.

The successful application of OGTAC-1 demonstrated the viability and generality of chemically induced O-GlcNAcylation in a dual fusion-protein system. We next pursued the induction of POI-specific O-GlcNAcylation by direct binding to the native domain of POIs; this could facilitate the induction of O-GlcNAcylation on endogenous POIs for functional dissection. To this end, we synthesized OGTAC-4, in which a JQ1^47^ structure was incorporated as a binding motif for the BRD4 BD1/BD2 domain (Figure 4A,B). As the N-terminal sequence of OGTAC-4 directly recruits BRD4, we first attempted to assess its potency on endogenous BRD4 in a cell line stably expressing fOGT. However, no O-GlcNAcylation signal for endogenous BRD4 could be detected by the IP-WB method using RL2 as the antibody (data not shown). We therefore overexpressed BRD4 together with fOGT in HEK293T cells to accommodate the readout within the IP-WB detection limit. In this coexpression system, we validated the O-GlcNAcylation-inducing effect of OGTAC-4 (Figure 4C). Of note, OGTAC-4 promoted BRD4 O-GlcNAcylation and the formation of a stable ternary complex between BRD4 and fOGT at concentrations as low as 3 nM (Figures 4C and S12). Compared with the covalent inducer, OGTAC-1 and OGTAC-4 demonstrated a wider effective concentration range. This may be attributed to the substoichiometric catalytic activity of OGTAC-4: even at lower concentrations, OGTAC-4 can remain effective because following the transfer of O-GlcNAc from fOGT to a BRD4 protein, the BRD4 dissociates from the original ternary complex, allowing for the recruitment of another unmodified BRD4 protein to fOGT. The kinetics of OGTAC-4-induced O-GlcNAcylation corresponds with this interpretation (Figure 4D). OGTAC-4 induced O-GlcNAcylation within 2 h, and the increased slope after 8 h indicates its induction has not saturated even after 24 h. Collectively, our results indicate that OGTAC-4 has catalytic characteristics and can be applied at low concentrations with long-lasting effects.

The O-GlcNAcylation sites on BRD4 have not been comprehensively studied and validated. We attempted to map out the O-GlcNAcylation sites on BRD4 induced by OGTAC-1/OGTAC-4 using IP-LC-MS/MS (Figure S13). Following the proteomics results, we conducted several site-directed mutagenesis studies, but immunoblot indicated that O-GlcNAc modification of BRD4 of the o-GlcNAc still existed after these mutations, suggesting that there are alternative modification sites (data not shown). We then truncated a fragment from the C-terminal of BRD4 (S1051 to F1224), where most high confidence O-GlcNAc sites were identified (Figure S20). In the absence of this fragment, no O-GlcNAc signal (RL2) can be detected following the treatment with OGTACs, and a weaker Co-IP of fOGT was observed. This indicates that this region is critical for OGT interaction and contains most of the O-GlcNAcylation sites (Figure 4E). Intensive mutagenesis and validation studies are required to specifically locate the O-GlcNAc sites on BRD4 in the future. Overall, we confirmed that the O-GlcNAcylation sites induced by OGTACs are located in the C-terminal region of BRD4, rather than in the HaloTag or as artifacts of immunoblotting. As this region encompasses the histone acetyltransferase activity domain (HAT) of BRD4, the impact of the O-GlcNAcylation on the HAT activity of BRD4 warrants further investigation in future studies.

### Enhancing Utility of the OGTAC Strategy by a Truncated fOGT Construct

**Figure 5:**
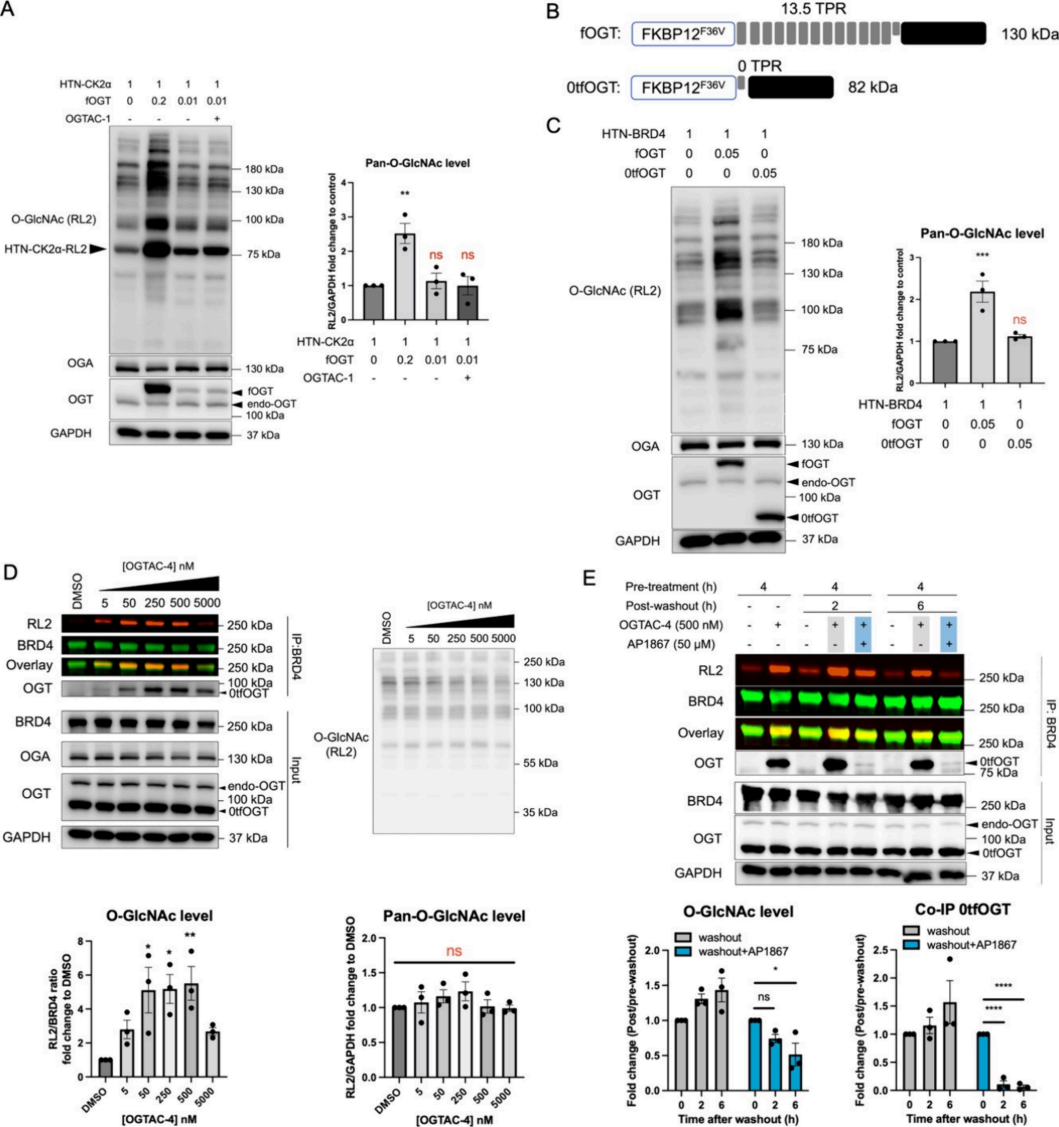
Specificity and reversibility of the OGTAC strategy. (A) Under the HTN-CK2α:fOGT = 1:0.01 cotransfection system, the cellular global O-GlcNAc level is not significantly changed with or without the treatment of the OGTAC-1 (125 nM). (B) Constructs of fOGT with 13.5 (rounded to 13) TPR and 0TPR-fOGT(0tfOGT); (C) 0tfOGT but not fOGT does not significantly change the cellular global O-GlcNAc level. (D) Dose-dependent O-GlcNAcylation profile of HTN-BRD4 by the OGTAC-4 treatment. HEK293T cells expressing HTN-BRD4:0tfOGT= 1:0.05 were treated with an increasing concentration of OGTAC-4 for 4 h. Then, the O-GlcNAcylation level of HTN-BRD4 was assessed by immunoblot after IP using the BRD4 antibody. All quantifications are shown as mean ± s.e.m. of *n* = 3 biologically independent experiments. (E) Reversible effect of OGTAC-4 on HTN-BRD4 can be achieved by addition of AP1867. HEK293T cells expressing HTN-BRD4:0tfOGT= 1:0.05 were pretreated with OGTAC-4 (500 nM) or DMSO for 4 h, and culturing media were replaced by fresh media with DMSO or AP1867 (50 μM) for the indicated time. The quantifications in right panel were conducted by the normalizing the fold-change (OGTAC-4/DMSO) for each postwashout time point to the value of 4 h pretreatment. All quantifications are shown as mean ± s.e.m. of 3 independent biological repeats. Statistical significance was calculated with ordinary one-way ANOVA comparing samples with different transfection conditions or OGTAC-treatment. ns, *p* ≥ 0.05; *p < 0.05; **p < 0.01; ***p < 0.001; ****p < 0.0001.

After illustrating the potency and generality of our OGTAC strategy, we then assessed its specificity toward the global O-GlcNAc proteome. We quantified the total pan-O-GlcNAc level (except for the specific band for target POI) using immunoblotting. For well-validated OGT substrates such as CK2α, a relatively low fOGT protein amount is sufficient to facilitate the inducing effect of our OGTAC-1. The pan-O-GlcNAc level did not significantly change under this condition (Figures 5A and S15); however, for BRD4, which is a controversial OGT substrate,^48^ the HTN-BRD4:fOGT = 1:0.01 condition is not sufficient for OGTAC to exert inducing effects even though the ternary complex was formed (Figure S14). We hypothesize that an optimal threshold of the OGT protein levels is required for efficient transfer of O-GlcNAc to BRD4. To address the trade-off between potency and specificity on BRD4, we developed a truncated fOGT system inspired by Walker’s and Woo’s previous studies.^17,49,50^ The tetratricopeptide repeat (TPR) domain of fOGT is responsible for substrate recognition; truncation of the TPR domain can reduce its activity toward protein substrates.^50^ We therefore constructed and compared several truncated fOGT (tfOGT) plasmids.

Among them, 0tfOGT showed minimum global O-GlcNAc level elevation even at the high expression level (Figures S16 and 5C). We next applied OGTAC-4 to the 0tfOGT:HTN-BRD4 system, resulting in a 5-fold increase in the level of the O-GlcNAc on BRD4. This result confirms that OGTAC-4 effectively leverages the activity of truncated fOGT to induce BRD4 O-GlcNAcylation without disruption of the global O-GlcNAc level (Figure 5D). Furthermore, we also verified that the inducing effect of OGTAC-4 is dependent on 0tfOGT (Figure S17).

To assess the potential application of our OGTAC strategy in downstream biological studies, we investigated the reversibility of the OGTACs. In a washout study using OGTAC-4 as an example, O-GlcNAc induction and ternary complex formation persisted postwashout, attributable to the high affinity of the JQ1 and AP1867 moieties for BRD4 and 0tfOGT, respectively. However, when AP1867 was introduced, a marked decrease in ternary complex formation occurred within 2 h, indicating that AP1867 could effectively disrupt the tight complex between 0tfOGT and BRD4 (Figure 5E). Additionally, the O-GlcNAc level of BRD4 returned to baseline after 6 h of AP1867 treatment, confirming the reversibility of the induction effect (Figure 5E). Notably, the global O-GlcNAc levels remained unchanged during the reversibility study (Figure S18).

We envision that this tfOGT system would be a versatile alternative method for inducing and investigating the O-GlcNAcylation of neo-substrates of OGT with high specificity, potency, and reversible control. However, OGT and its truncated variants have been reported to exhibit varied O-GlcNAcylation site selection on substrates.^50^ Therefore, future studies should concentrate on conducting side-by-side proteomics analyses to compare the site specificity between fOGT and tfOGT on the POIs.

## Conclusions

Here, we developed a chemogenetic technology, namely, OGTAC, by harnessing the promiscuity of the OGT for inducing protein-specific O-GlcNAcylation in living cells. OGTAC-1 is a chemical inducer of O-GlcNAcylation generally applicable for tagged POIs, as shown here by BRD4, CK2α, and EZH2. In addition, OGTAC-4 was developed for induction of BRD4-specific O-GlcNAcylation. Both OGTAC-1 and OGTAC-4 showed submicromolar O-GlcNAcylation inducing activity. OGTAC-1 covalently bound the POIs and demonstrated an obvious hook effect at higher concentrations, while OGTAC-4 functioned as a substoichiometric catalyst for BRD4-specific O-GlcNAcylation, therefore inducing an effect across a wider concentration range. To optimize the OGTAC strategy for biological investigations, we recommend coexpressing truncated fOGT (0tfOGT) with proteins of interest at a POI:0tfOGT ratio of 1:0.05 to minimize perturbation of global O-GlcNAc levels. Reversibility of the induced O-GlcNAcylation can be achieved through the washout of the OGTACs plus the addition of AP1867.

Despite the growing recognition of the significant roles that protein-specific O-GlcNAcylation plays in various cellular processes and diseases, methods to uncover these roles remain underdeveloped. Genetic mutation of glycosites^44,51,52^ can help elucidate the site-specific function of O-GlcNAcylation, but this approach has limitations: (1) it may alter protein folding and substrate recognition and interfere with other PTMs such as phosphorylation; (2) it lacks the necessary temporal and magnitude control due to the dynamic and substoichiometric nature of O-GlcNAcylation. Although nanobody-OGT^17^ and dual-specific RNA aptamers^16^ have been recently developed, their mechanisms inevitably constrain them from precise temporal and magnitude control. Our OGTAC technology provides an alternative option for inducing POI-specific O-GlcNAcylation. While excessive OGT-POI binding could interfere with native protein–protein interactions (PPIs) and hinder subsequent functional studies, the rapid kinetics and reversibility of the OGTAC offer notable advantages. Specifically, POIs can be swiftly O-GlcNAcylated following the OGTAC treatment, and the application of excess AP1867 can release POIs from their tight complexes with tfOGT, thereby minimizing interference in downstream studies.

During the development of the OGTAC technology, we noticed that linker length is critical for optimal transfer of the O-GlcNAc from the OGT to POIs. Target engagement assays and evaluations of O-GlcNAcylation-inducing effects of the O-GlcNAcylation-inducing reaction revealed that OGTAC-2 and 3, both with longer linkers, effectively promote the formation of ternary complexes but are less efficient at inducing the O-GlcNAcylation than OGTAC-1. This suggests that longer linkers may impede the O-GlcNAc transfer from OGT to POIs (Figure S19). The tetratricopeptide repeat (TPR) domain of OGT typically requires close proximity to its substrate for recognition, underscoring the importance of physical distance.^53,54^ Extended flexible linkers not only increase the physical distance between the OGT and POIs, but also lead to more complex protein dynamics, which may weaken PPIs. Consequently, we propose that OGTAC-1 induced the optimal distance and conformation between the OGT catalytic site and POIs. In contrast, OGTAC-2 and 3, despite promoting complex formation via fused tags, may not favor productive PPI due to their extended distance and flexible linker structure, which could be detrimental to optimal O-GlcNAc transfer (Figure S19).

Although we have successfully achieved targeted O-GlcNAcylation using bifunctional molecules, limitations remain in the approach. Specifically, it necessitates the exogenous coexpression of POIs and fOGT/0tfOGT. This is partly due to the typically low O-GlcNAcylation levels of endogenous POIs that fall below detection limits and the absence of a noninhibitory ligand of native OGT. Identifying a binder capable of recruiting endogenous OGT would mark a significant advancement. Developing bifunctional molecules, or a “molecular glue” variant of OGTACs, could facilitate O-GlcNAcylation of endogenous POIs, thereby overcoming current challenges such as reliance on plasmid cotransfection efficiency and the potential trade-off in the site-selectivity of 0tfOGT.^49,50^

In our laboratory, we are currently exploring the functional roles of POI-specific O-GlcNAcylation by utilizing OGTACs. The rapid kinetics and reversibility of the OGTAC strategy enable in-depth investigations into the roles of O-GlcNAcylation in various dynamic signaling pathways. Furthermore, methods to detect and quantify protein-specific O-GlcNAcylation remain to be improved, with quantitative proteomics being indispensable to accurately determining the status of the O-GlcNAcylation at the site-specific level.

Our OGTAC technology demonstrates the potential of using small molecules to induce protein-specific O-GlcNAcylation, providing a novel tool for more deeply understanding the functional roles of protein O-GlcNAcylation. Additionally, it lays the foundation for therapeutically targeting dysregulated O-GlcNAcylation in human diseases.

## Supporting Information Available

Methods, supplementary figures, organic synthesis, and characterization data (PDF)
Analyzed proteomics data (XLSX)
