# Supplementary material for: Targeted Protein O-GlcNAcylation Using Bifunctional Small Molecules
Source: J Am Chem Soc. 2024 Apr 1;146(14):9779–89. doi: 10.1021/jacs.3c14380 (PMC11009946; doi:10.1021/jacs.3c14380)
Supplement: Supplementary file 1 — ja3c14380_si_001.pdf [file ja3c14380_si_001.pdf]

Supplementary Information

Targeted protein O-GlcNAcylation using bifunctional small molecules

Bowen Ma[a#], Khadija Shahed Khan[a, b#], Tongyang Xu[a], Josefina Xeque Amada[a], Zhihao Guo[a], Yunpeng Huang[a], Yu Yan[a], Henry Lam[d], Alfred Sze-Lok Cheng[b], Billy Wai-Lung Ng[a,c*]

[a] School of Pharmacy, Faculty of Medicine, The Chinese University of Hong Kong, Hong Kong
[b] School of Biomedical Sciences, Faculty of Medicine, The Chinese University of Hong Kong, Hong Kong
[c] Li Ka Shing Institute of Health Sciences, Faculty of Medicine, The Chinese University of Hong Kong, Hong Kong
[d]Department of Chemical and Biological Engineering, The Hong Kong University of Science and Technology, Hong Kong

Supplementary figures:

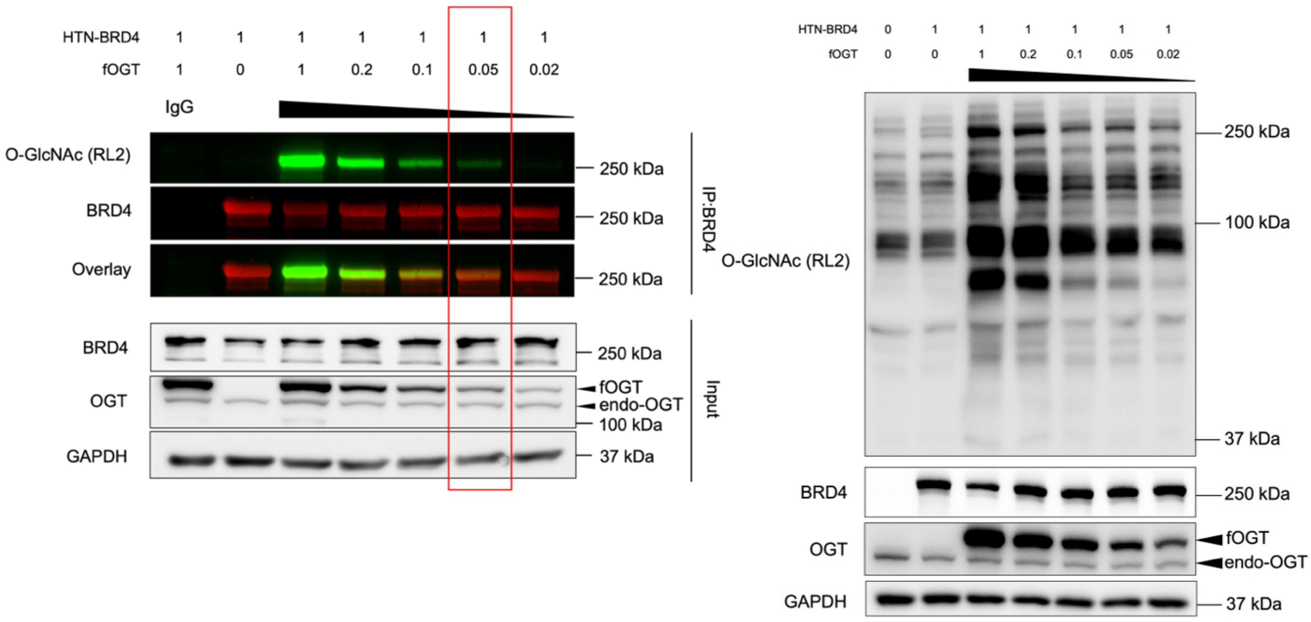

Figure S1. HTN-BRD4:fOGT plasmid ratio optimisation. Co-transfection of 5 µg pHTN-BRD4 with 0.05 µg fOGT (1:0.05) (red) was selected for later study. The co-transfection effect on pan-O-GlcNAc is shown in the right panel.

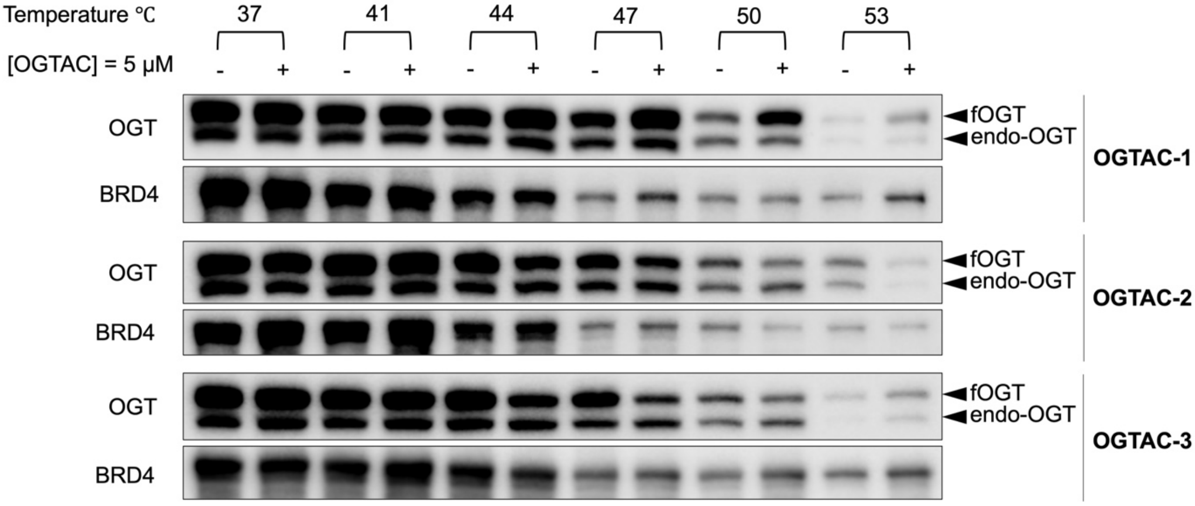

Figure S2. Representative immunoblots analysis for CETSA experiments. All probes were treated at 5 µM for 4 h in HEK293T cells expressing HTN-BRD4:fOGT= 1: 0.05.

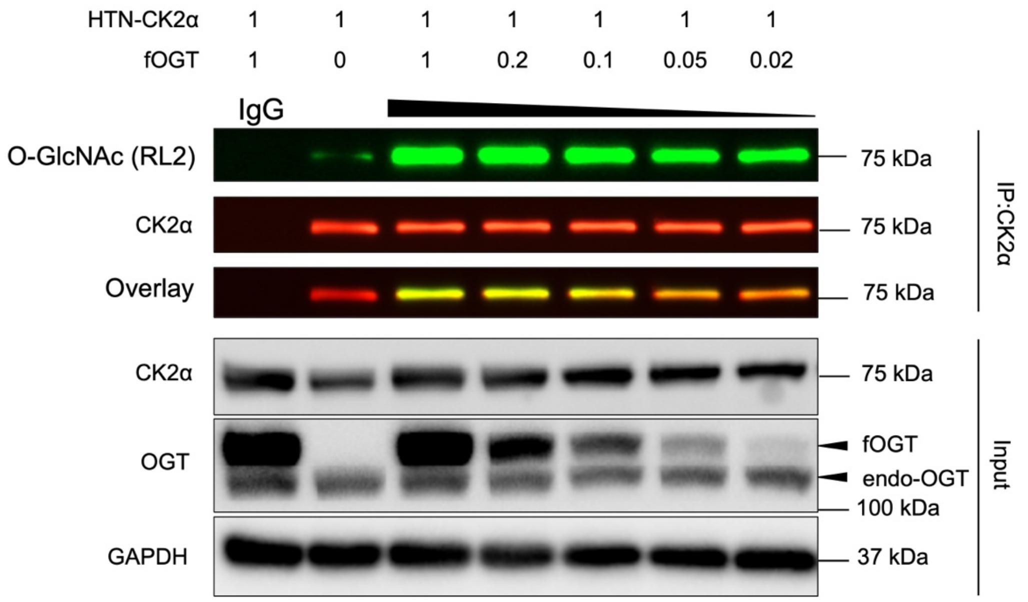

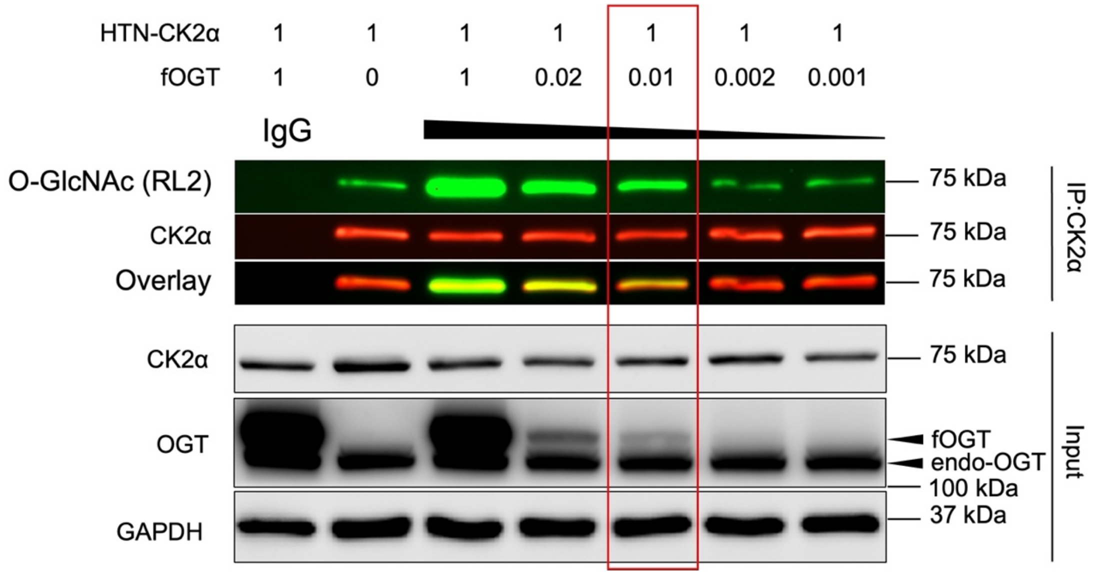

Figure S3. HTN-CK2α:fOGT plasmid ratio optimisation. Co-transfection of 5 μg pHTN-CK2α with 0.05 μg fOGT (1:0.01) (red) was selected for later study.

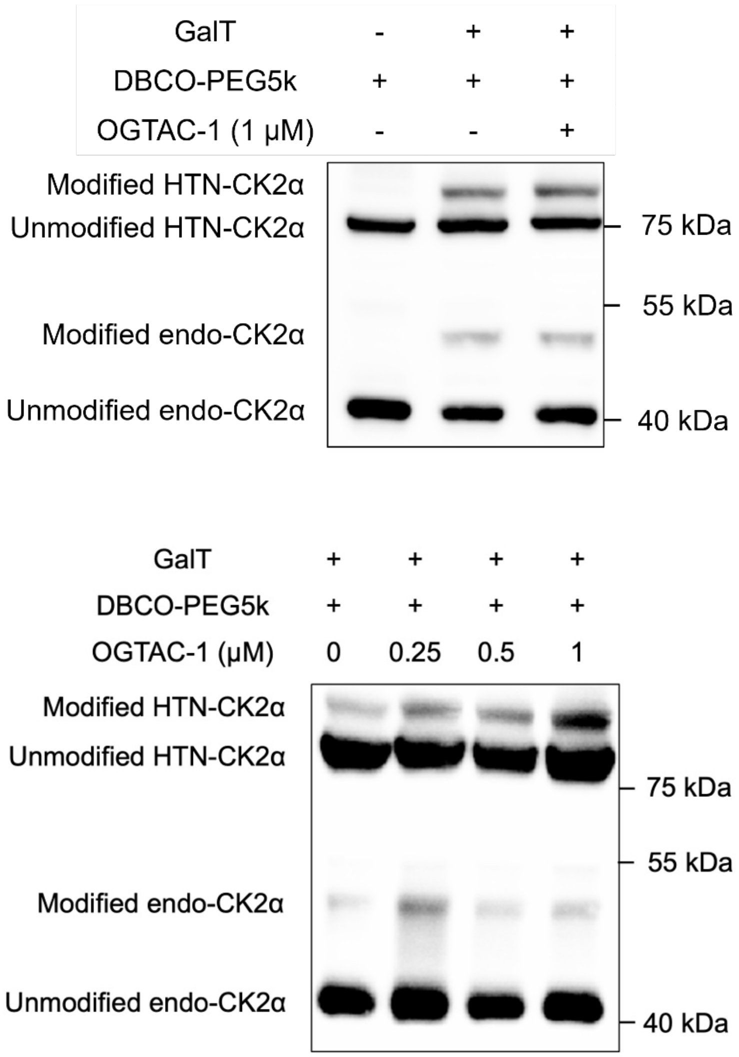

Figure S4. Biological repeats of GalT based mass-shift assay to validate the OGTAC-1 induced HTN-CK2α specific O-GlcNAcylation.

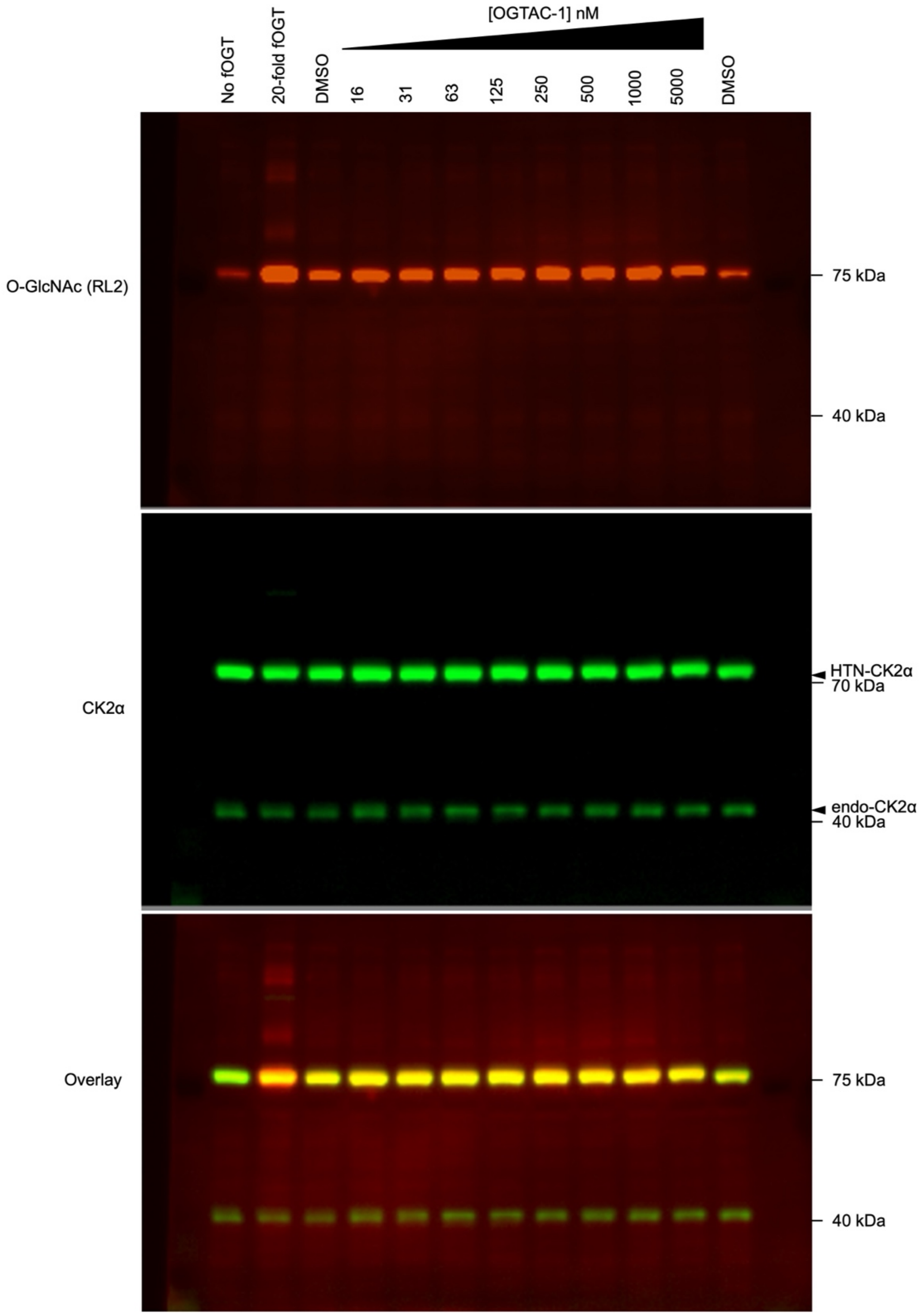

Figure S5. Evaluation of HTN-CK2α O-GlcNAc level from whole cell lysate WB. Overlapping the pan-RL2 (red) with HTN-CK2α (green at ~75 kDa) reveals the HTN-CK2α specific O-GlcNAc level. 20-fold fOGT was used as positive control, with a significantly induced O-GlcNAcylation level.

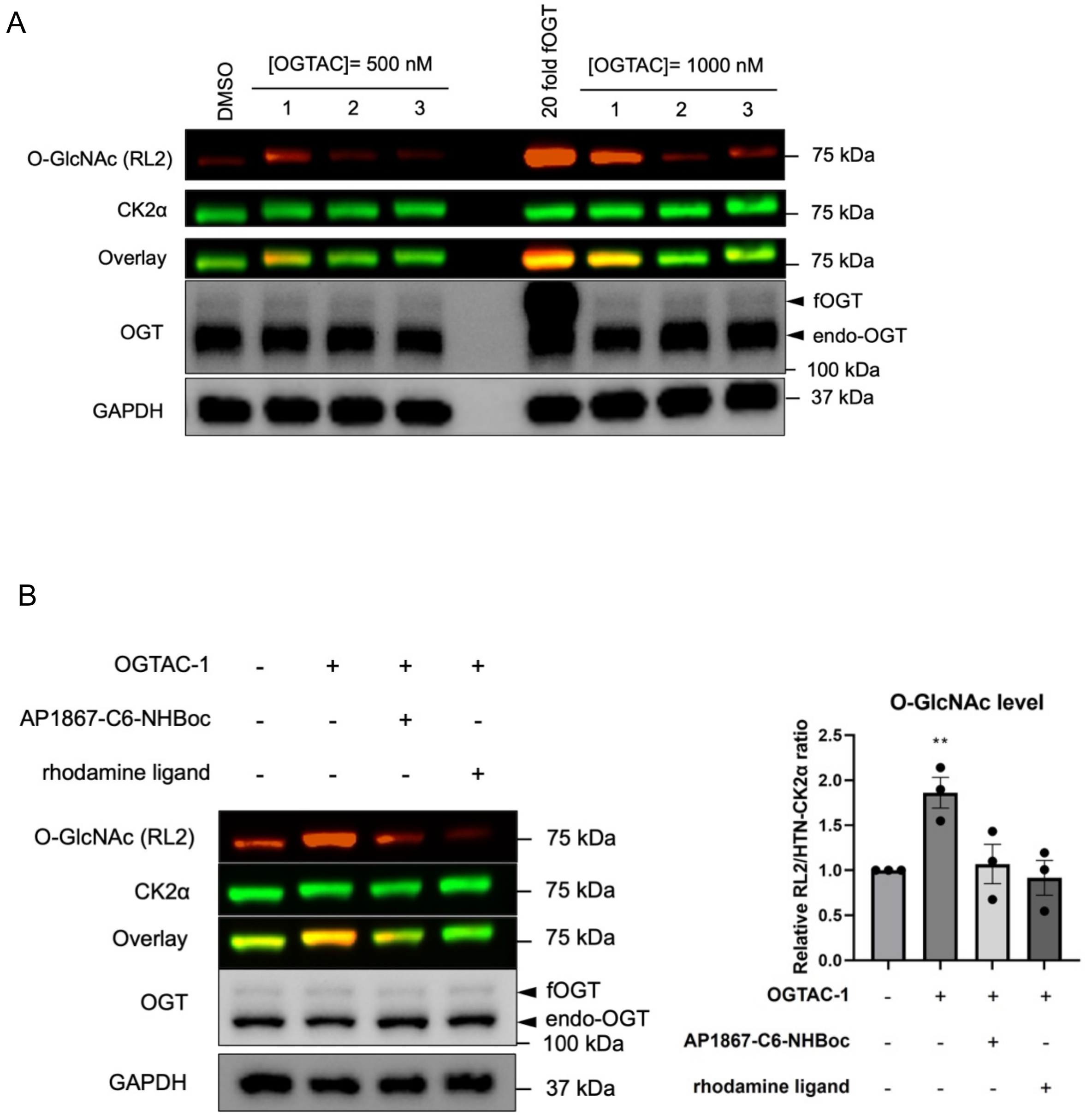

Figure S6. Evaluation of O-GlcNAc inducing effects of OGTACs on HTN-CK2α from whole cell lysate WB. (A), OGTAC-1 induced higher fold increase of O-GlcNAcylation on HTN-CK2α in both concentration at 500 nM and 1 μM; (B), Immunoblot analysis of OGTAC-1 mediated O-GlcNAcylation when co-treated with 10x concentration of binding competitors. All quantifications are shown as mean ± s.e.m. of 3 biologically independent repeats. Statistical significance was calculated with unpaired two-tailed Student's t tests comparing DMSO- to OGTAC-1- treated samples. **p < 0.01.

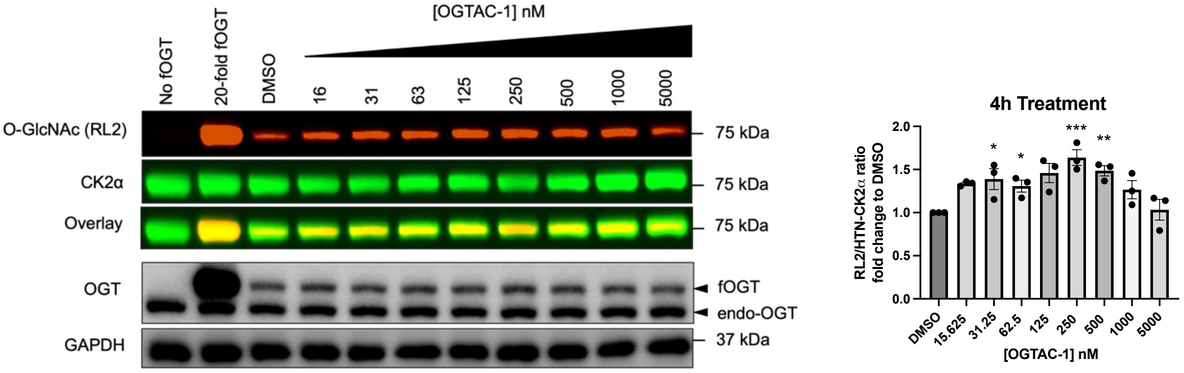

Figure S7. OGTAC-1 dose-dependent O-GlcNAc inducing effect on HTN-CK2α after 4 h treatment. The quantification was conducted by immunoblot signal of RL2 relative to HTN-CK2α as the mean ± s.e.m. of n = 3 biologically independent experiments. Statistical significance was calculated with ordinary one-way ANOVA comparing DMSO- to OGTAC-1-treated samples. *p < 0.05, **p < 0.01, ***p < 0.001.

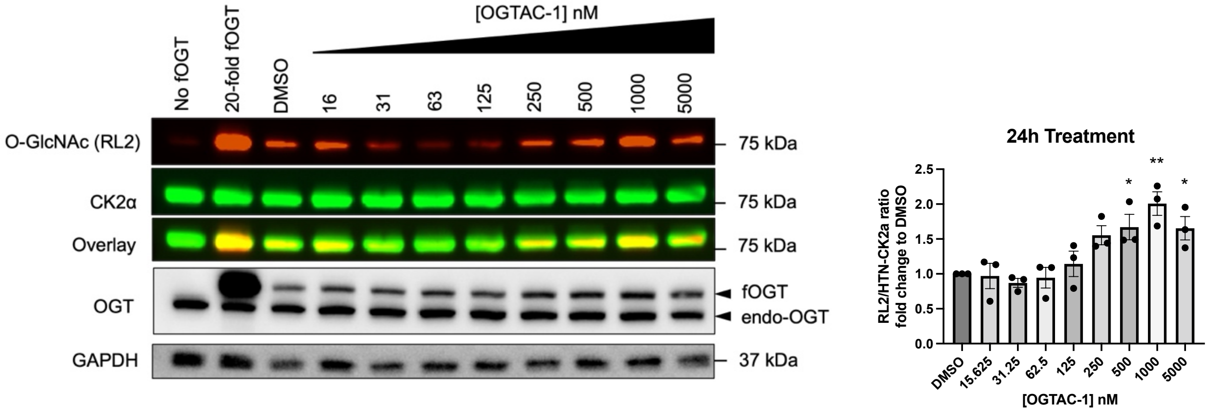

Figure S8. OGTAC-1 dose-dependent O-GlcNAc inducing effect on HTN-CK2α after 24 h treatment. The quantification was conducted by immunoblot signal of RL2 relative to HTN-CK2α as the mean ± s.e.m. of n = 3 biologically independent experiments. Statistical significance in was calculated with ordinary one-way ANOVA comparing DMSO- to OGTAC-1-treated samples. *p < 0.05, **p < 0.01.

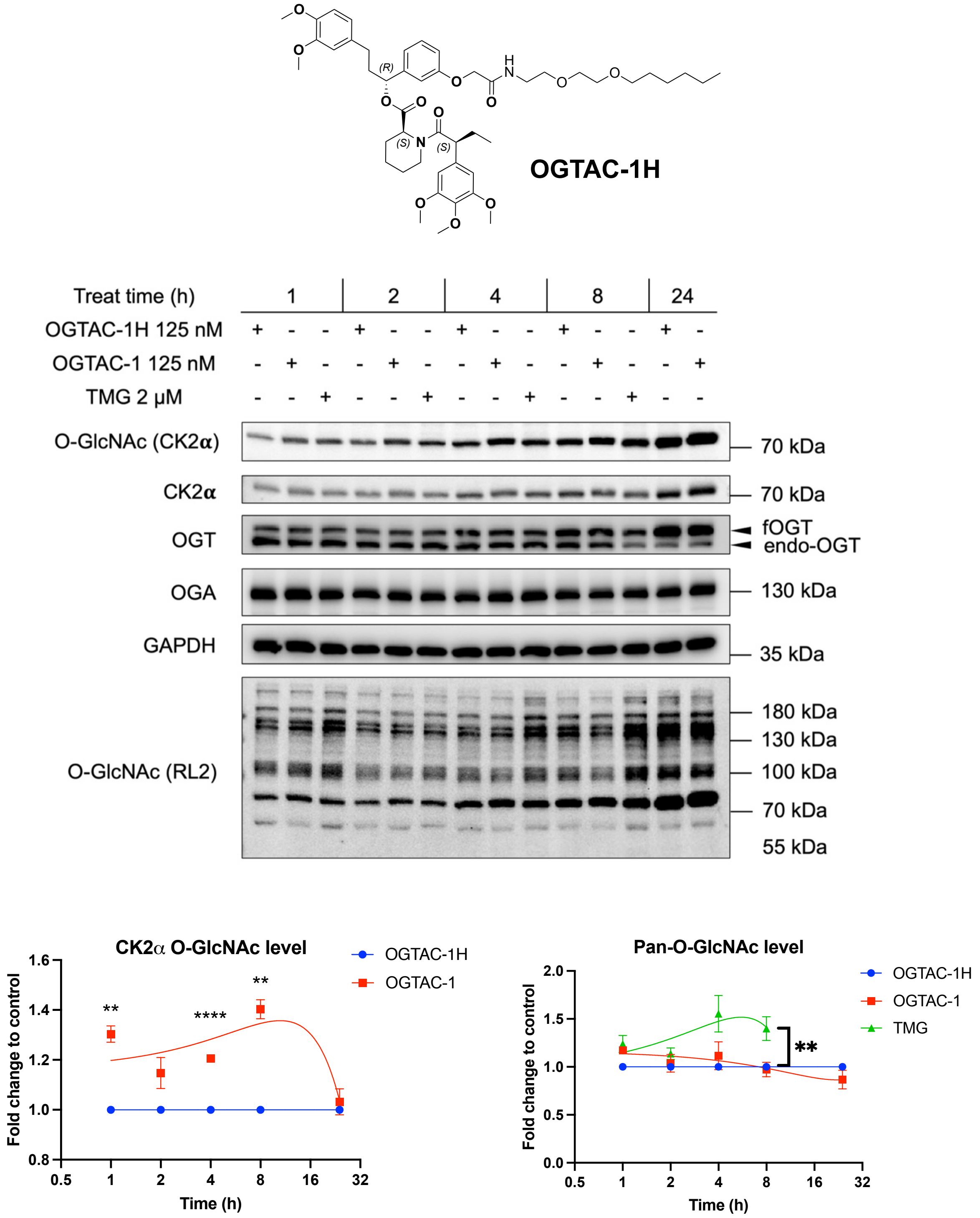

Figure S9. Immunoblotting analysis of time-dependent effects of OGTAC-1 on HTN-CK2α. OGTAC-1H was an inactive, negative control molecule of OGTAC-1. The quantifiation was conducted by immunoblot signal of total RL2 (except the band specific for HTN-CK2α) relative to GAPDH as the mean ± s.e.m. of n = 3 biologically independent experiments. Statistical significance was calculated by multiple unpaired t-tests. ns, **p < 0.01, ****p < 0.0001.

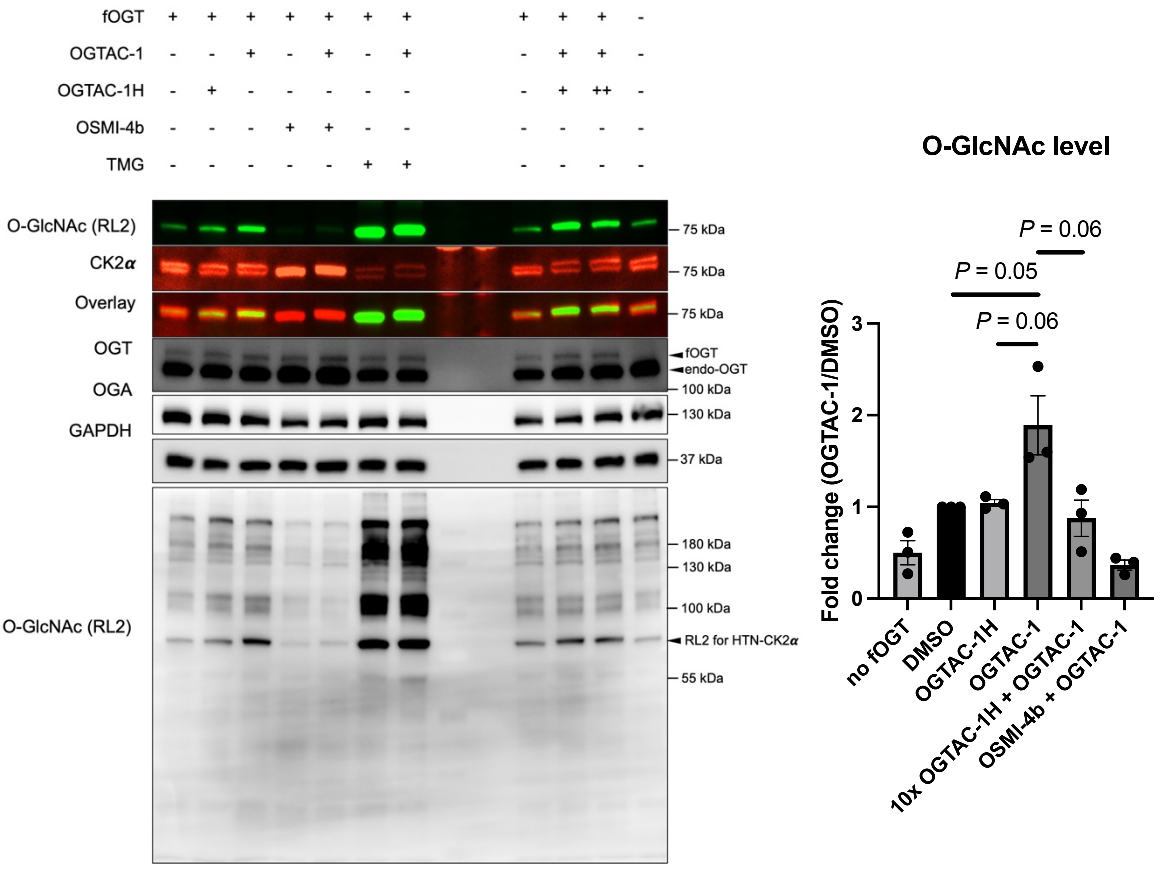

Figure S10. Immunoblotting analysis of effects of OGTAC-1 on HTN-CK2α in HeLa cell line. HeLa cells were transfected with fOGT:HTN-CK2α=1:0.1 for 24 h, followed by treatment of OGTAC-1 (125 nM) or other probes for 8 h. For OGTAC-1H, ‘++’ denotes 10-fold concentration of OGTAC-1, while “+” denote the same concentration of OGTAC-1. The quantification was conducted by immunoblot signal of RL2 relative to CK2α as the mean ± s.e.m. of n = 3 biologically independent experiments. Statistical significance was calculated by unpaired t-tests.

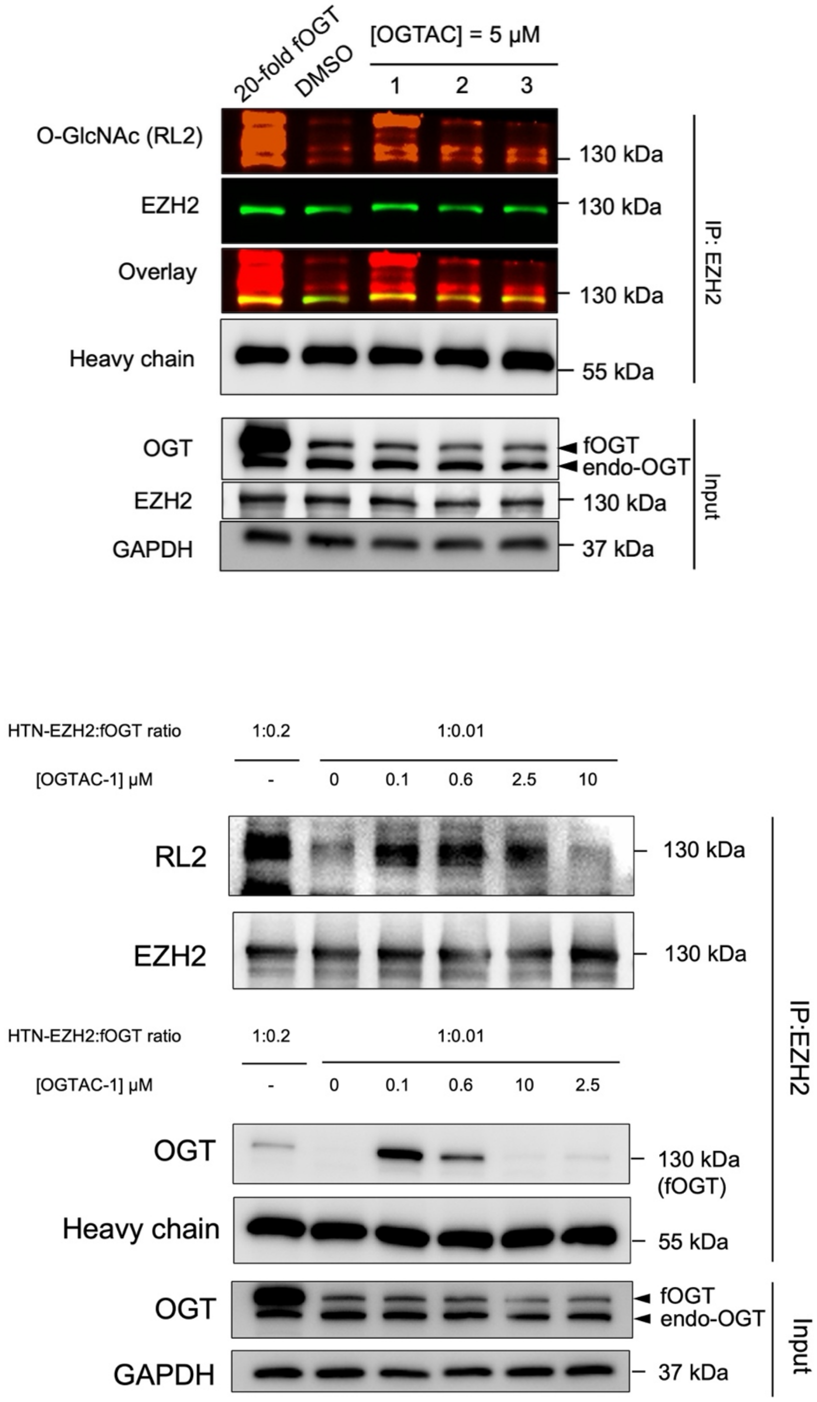

Figure S11. Evaluation of O-GlcNAc inducing effects of OGTAC-1/2/3 on HTN-EZH2 and dose-dependent effect of OGTAC-1 on HTN-EZH2. In the treatment group, transfection ratio is HTN-EZH2:fOGT: = 1:0.01.

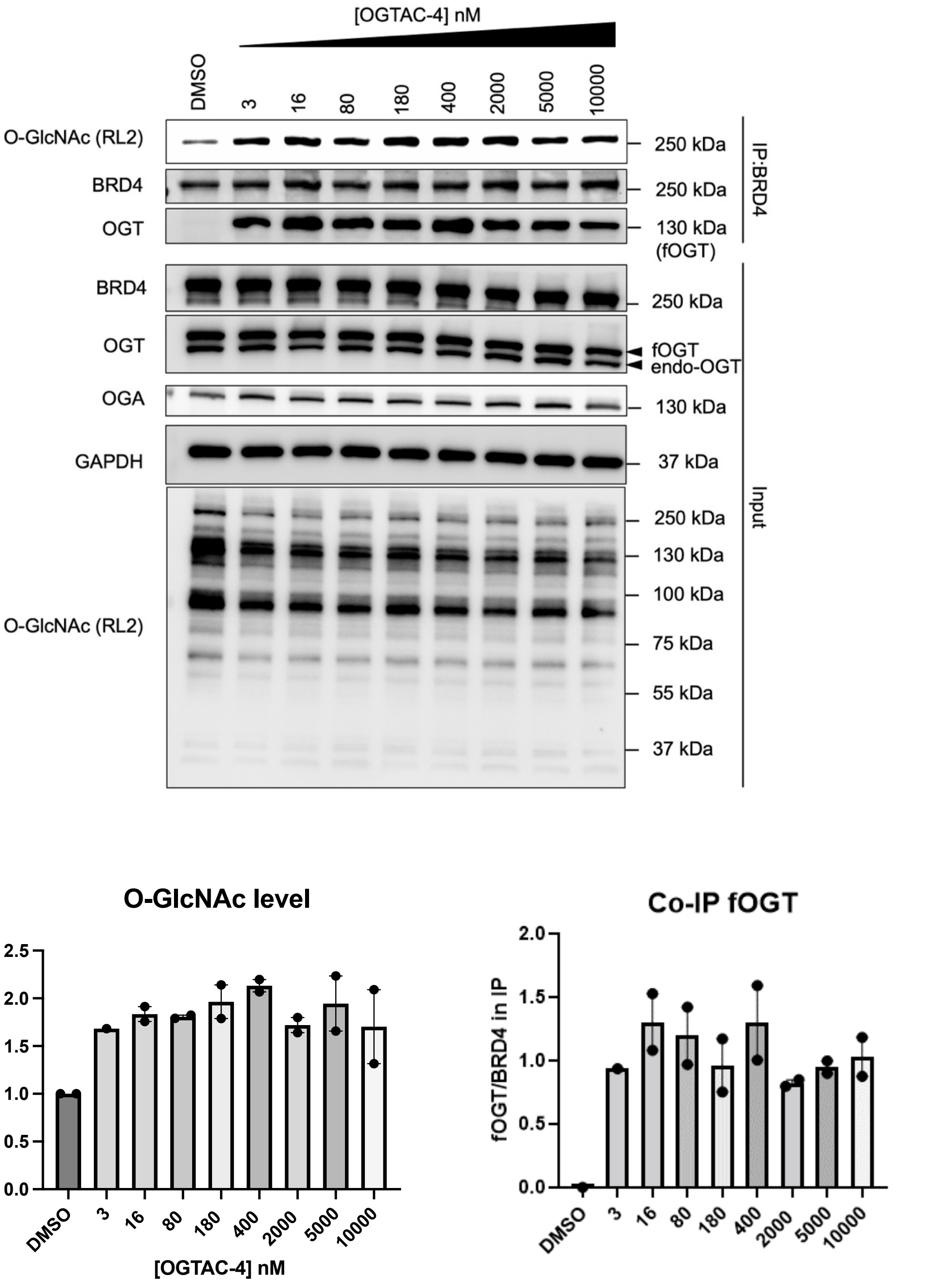

Figure S12. Evaluation of O-GlcNAc inducing effects at wider concentration range of OGTAC-4 on HTN-BRD4 by IP-WB method. The quantification was conducted by immunoblot signal of RL2/HTN-BRD4 as the mean ± s.e.m. of n = 2 biologically independent experiments. For 3 nM of OGTAC-4, the experiment was conducted once.

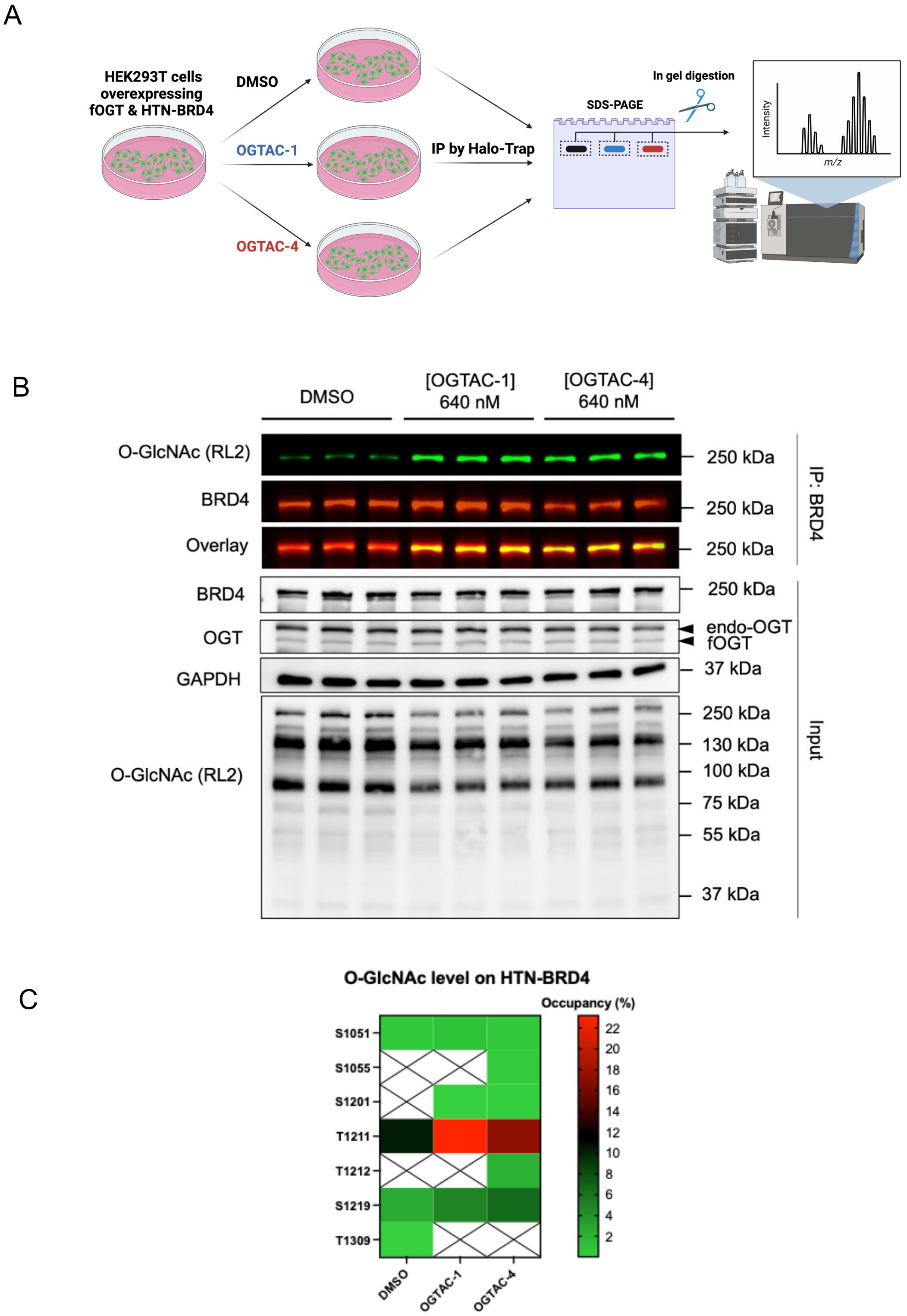

Figure S13. Evaluation of O-GlcNAc inducing effects of OGTAC-1 and OGTAC-4 on HTN-BRD4 using proteomics. (A), Cells treated with DMSO, OGTAC-1 (640 nM), or OGTAC-4 (640 nM) for 4 h were lysed, enriched by Halo-Trap then submitted to WB (B). Same samples were separated by SDS-PAGE, the gel bands for HTN-BRD4 were cut, trypsinized for quantitative shotgun proteomics. The occupancy of each O-GlcNAcylated sites upon different treatment were shown in the heatmap (C). Analyzed results in excel files were attached as separate files (XLSX).

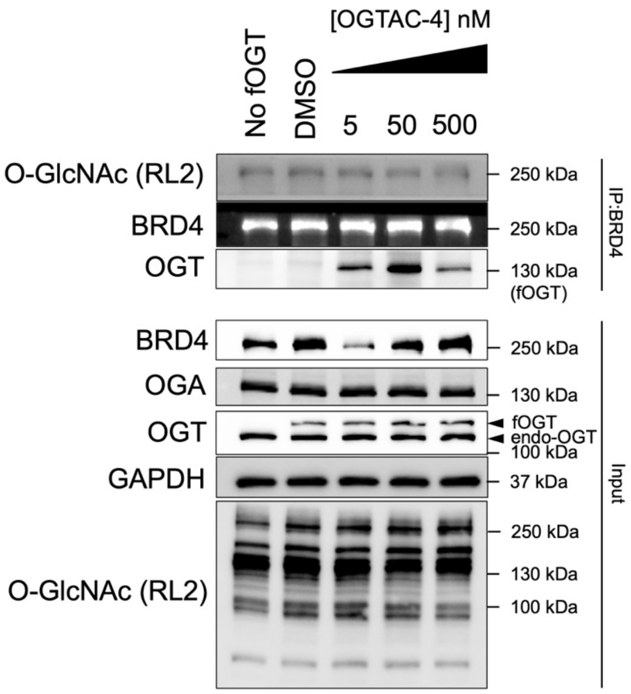

Figure S14. Immunoblotting analysis of OGTAC-4 inducing effect in HTN-BRD4:fOGT =1:0.01 system. No O-GlcNAcylation inducing effect of OGTAC-4 on HTN-BRD4 was observed.

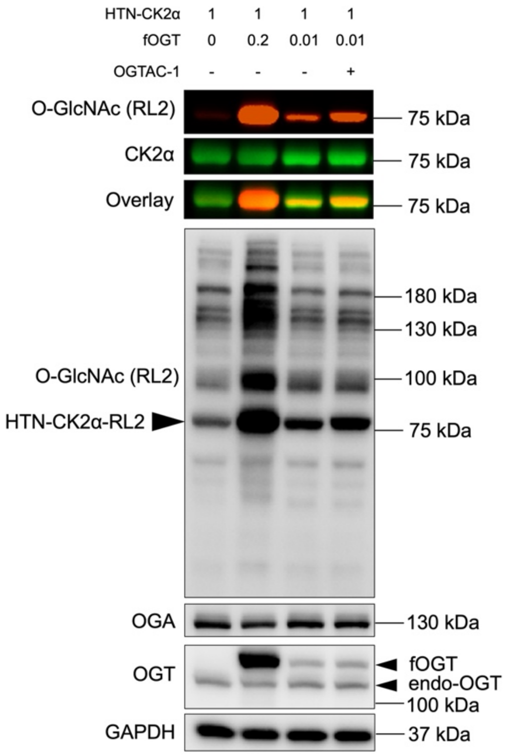

Figure S15. Immunoblotting analysis of the specificity of our chemogenetic system with HTN-CK2α:fOGT=1:0.01. OGTAC-1 was used at 125 nM.

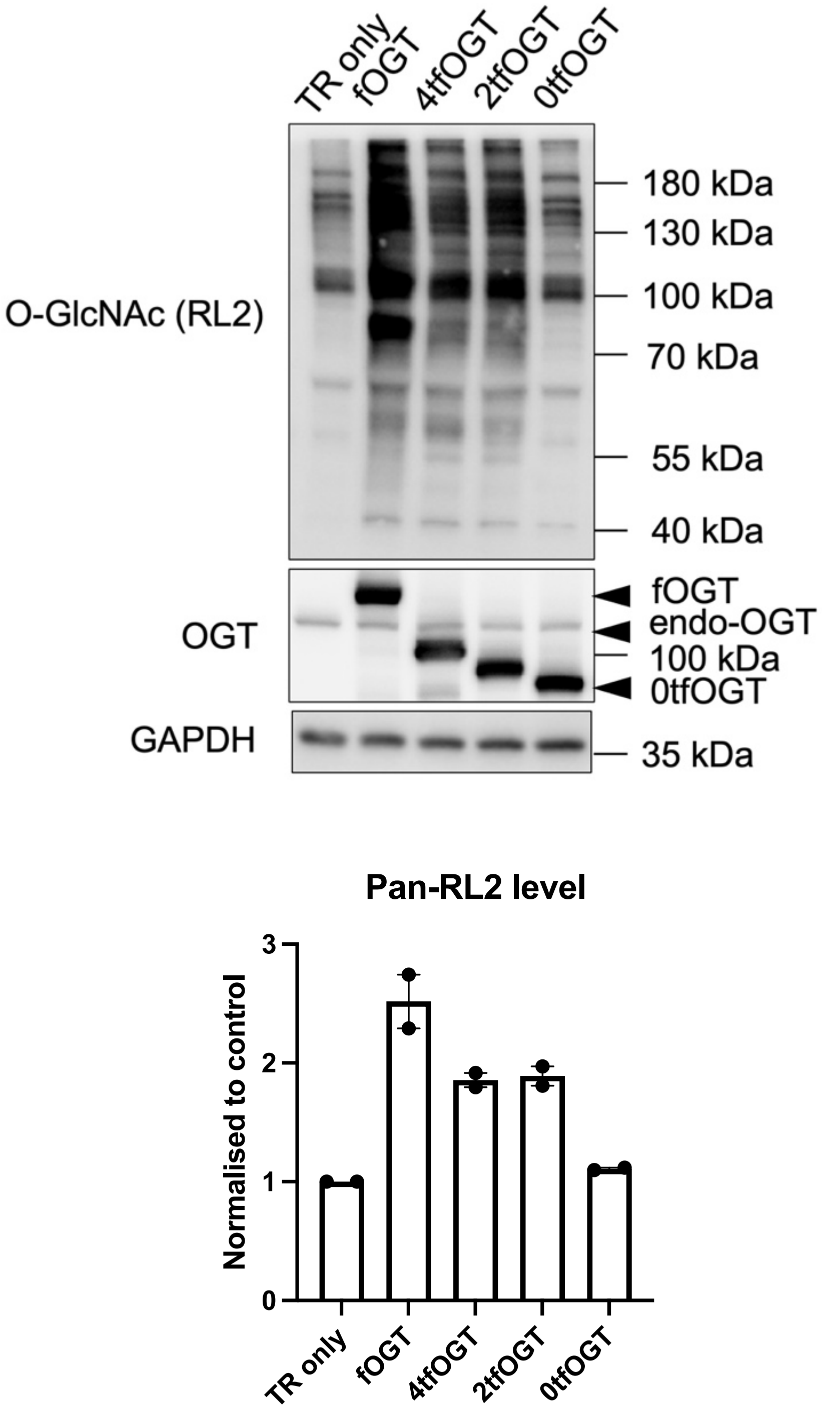

Figure S16. Immunoblotting analysis of the O-GlcNAcylation inducing effect of truncated fOGT (tfOGT) on global celluar proteins. The quantification was conducted by immunoblot signal of pan-RL2/GAPDH as the mean ± s.e.m. of n = 2 biologically independent experiments. TR, transfection reagent.

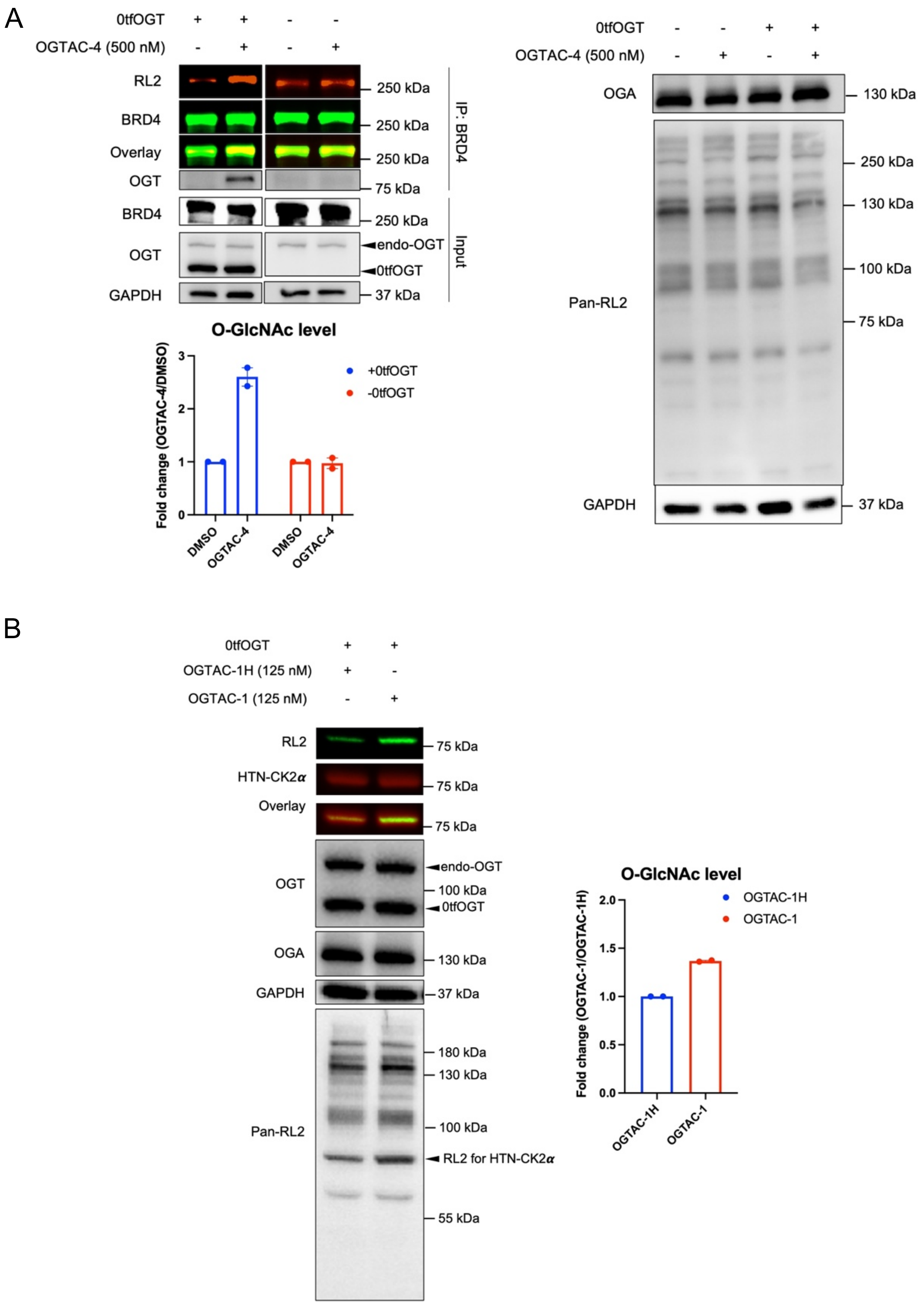

Figure S17. Immunoblotting analysis of the global O-GlcNAcylation inducing effect of truncated OGTAC-4 and OGTAC-1 on HTN-BRD4 and HTN-CK2α respectively. (A), O-GlcNAcylation inducing effect of OGTAC-4 is dependent on the expression of 0tfOGT; (B), OGTAC-1 induced higher O-GlcNAcylation level comparing to the negative control OGTAC-1H. The chlorine-to-hydrogen substitution renders OGTAC-1H unable to recruit 0tfOGT to HTN-CK2α, suggesting that the inducing effect is 0tfOGT-dependent. The quantification was conducted by immunoblot signal of probe treatment/control as the mean ± s.e.m. of n = 2 biologically independent experiments. TR, transfection reagent.

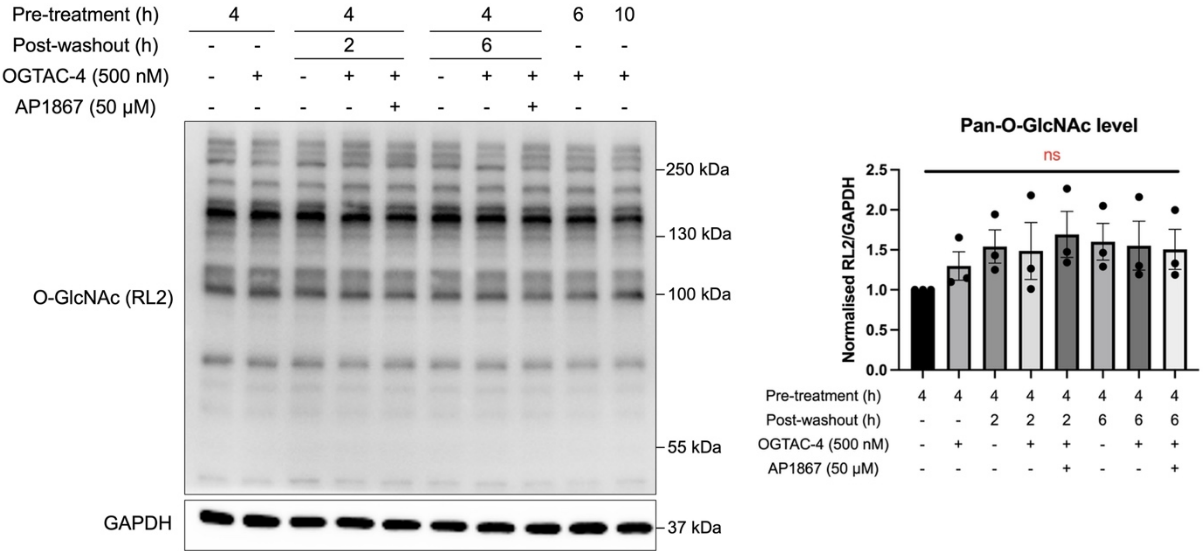

Figure S18. Immunoblotting analysis of global O-GlcNAcylation level during reversibility study. The quantification was calculated by immunoblot signal of total RL2 relative to GAPDH as the mean ± s.e.m. of n = 3 biologically independent experiments. Statistical significance was calculated by multiple unpaired t-tests. ns, not significant.

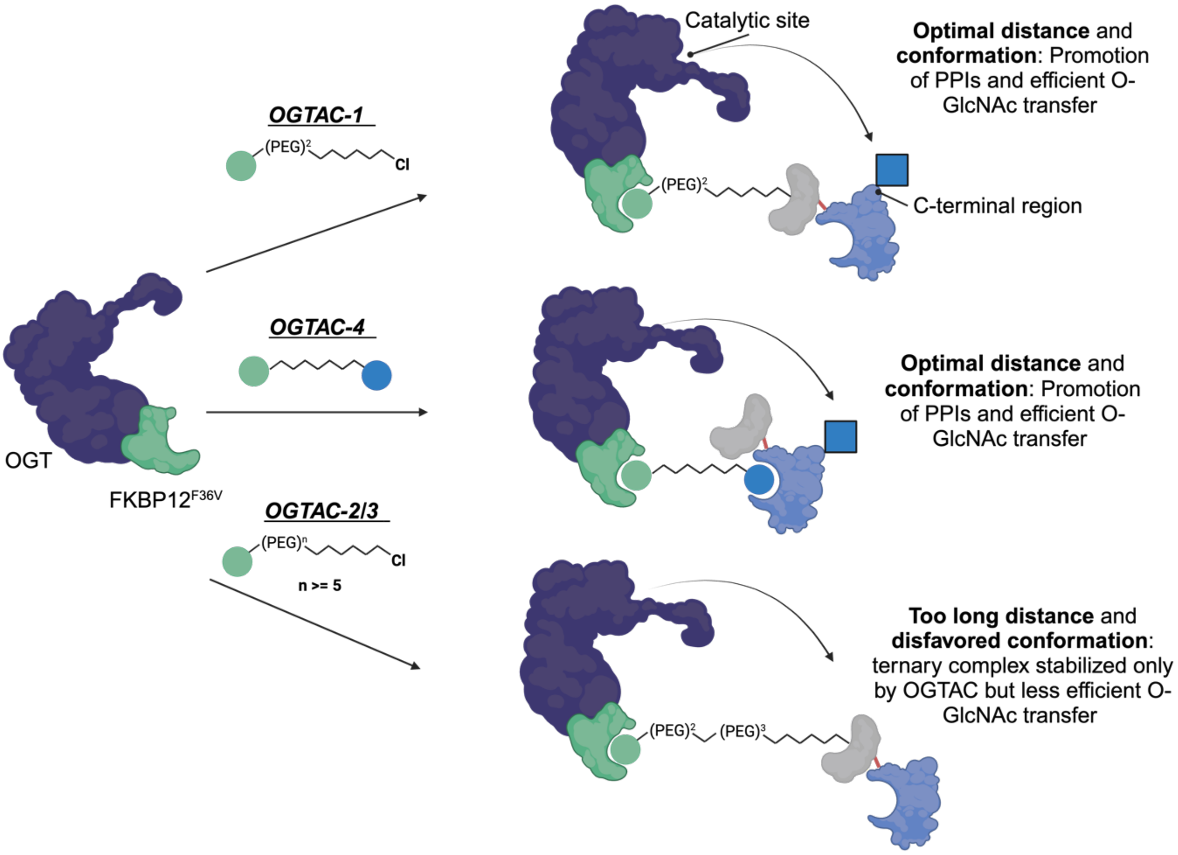

Figure S19. Proposed rationale for the activity difference between OGTACs and why stable ternary complex does not mean efficient induction of O-GlcNAcylation.

HTN-BRD4 sequence:

MAEIGTGFPFDPHYVEVLGERMHYVDVGPRDGTPVLFLHGNPTSSYVWRNIIPHVA
PTHRCIAPDLIGMGKSDKPDLGYFFDDHVRFMDAFIEALGLEEVVLVIHDWGSALGF
HWAKRNPERVKGIAFMEFIRPIPTWDEWPEFARETFQAFRTTDVGRKLIIDQNVFIE
GTLPMGVVRPLTEVEMDHYREPFLNPVDREPLWRFPNELPIAGEPANIVALVEEYM
DWLHQSPVPKLLFWGTPGVLIPPAEAARLAKSLPNCKAVDIGPGLNLLQEDNPDLIG
SEIARWLSTLEISGEPTTEDLYFQSDNAIASEFMSAESGPGTRLRNLPVMGDGLETS
QMSTTQAQAQPQPANAASTNPPPPETSNPNKPKRQTNQLQYLLRVVLKTLWKHQ
FAWPFQQPVDAVKLNLPDYYKIIKTPMDMGTIKKRLENNYYWNAQECIQDFNTMFT
NCYIYNKPGDDIVLMAEALEKLFLQKINELPTEETEIMIVQAKGRGRGRKETGTAKP
GVSTVPNTTQASTPPQTQTPQPNPPPVQATPHPFPAVTPDLIVQTPVMTVVPPQPL
QTPPPVPPQPQPPPAPAPQPVQSHPPIIAATPQPVKTKKGVKRKADTTTPTTIDPIH
EPPSLPPEPKTTKLGQRRESSRPVKPPKKDVPDSQQHPAPEKSSKVSEQLKCCSG
ILKEMFAKKHAAYAWPFYKPVDVEALGLHDYCDIIKHPMDMSTIKSKLEAREYRDAQ
EFGADVRLMFSNCYKYNPPDHEVVAMARKLQDVFEMRFAKMPDEPEEPVVAVSS
PAVPPPTKVVAPPSSSDSSSDSSSDSDSSTDDSEEERAQRLAELQEQLKAVHEQL
AALSQPQQNKPKKKEKDKKEKKKEKHKRKEEVEENKKSKAKEPPPKKTKKNNSSN
SNVSKKEPAPMKSKPPPTYESEEEDKCKPMSYEEKRQLSLDINKLPGEKLGRVVHII
QSREPSLKNSNPDEIEIDFETLKPSTLRELERYVTSCLRKKRKPQAEKVDVIAGSSK
MKGFSSSESESSSESSSSDSEDSETEMAPKSKKKGHPGREQKKHHHHHHQQMQ
QAPAPVPQQPPPPPQQPPPPPPPQQQQPPPPPPPPSMPQQAAPAMKSSPPPFI
ATQVPVLEPQLPGSVFDPIGHFTQPILHLPQPELPPHLPQPPEHSTPPHLNQHAVVS
PPALHNALPQQPSRPSNRAAALPPKPARPPAVSPALTQTPLLPQPPMAQPPQVLLE
DEEPPAPPLTSMQMQLYLQQLQKVQPPTPLLPSVKVQSQPPPPLPPPPHPSVQQQ
LQQQPPPPPPPQPQPPPQQQHQPPPRPVHLQPMQFSTHIQQPPPPQGQQPPHP
PPGQQPPPPQPAKPQQVIQHHHSPRHHKSDPYSTGHLREAPSPLMIHSPQMSQF
QSLTHQSPPQQNVQPKKQELRAASVVQPQPLVVVKEEKIHSPIIRSEPFSPSLRPEP
PKHPESIKAPVHLPQRPEMKPVDVGRPVIRPPEQNAPPPGAPDKDKQKQEPKTPV
APKKDLKIKNMGSWASLVQKHPTTPSSTAKSSSDSFEQFRRAAREKEEREKALKA
QAEHAEKEKERLRQERMRSREDEDALEQARRAHEEARRRQEQQQQRQEQQQ
QQQQQAAAVAAAATPQAQSSQPQSMLDQQRELARKREQERRRREAMAATIDMN
FQSDLLSIFEENLF

Figure S20. Construct of HTN-BRD4 used in this study. HaloTag fragment was labelled in purple. The 1051-1224 fragment (where most O-GlcNAc modification occurs) was highlighted in red.

Materials and Methods:

Cell Cultures, DNA Constructs, and Reagents

HEK293T cells and HeLa cells (kind gift from Prof. Sze Lok Cheng, CUHK) were maintained and cultured at 37 °C with 5% CO_2 in Dulbecco's modified Eagle's medium (DMEM) supplemented with 10 % fetal bovine serum and 1% penicillin and streptomycin.

FKBP12^{F36V}-OGT vector was a gift from Walker's group (Harvard Medical School).[1]

HTN-BRD4 plasmids were constructed by subclone from pcDNA4-TO-HA-Brd4FL (addgene: #31351), then homologous recombination (Vazyme C115) into pHTN HaloTag® CMV-neo Vector (Promega G7711).

HTN-CK2α plasmids were constructed by subclone from pDB1 (CK2αlpha) (addgene: #27083), then homologous recombination into pHTN HaloTag® CMV-neo Vector.

HTN-EZH2 plasmids were constructed by subclone from 3XMyc-His$_6$-EZH2 plasmid from Prof. YangChao Chen (CUHK), then homologous recombination into pHTN HaloTag® CMV-neo Vector.

Transient transfection was conducted according to the manufacture's protocol using Lip2000 TR (AboRo, RL0401).

Plasmids site-directed mutagenesis was conducted according to the manufacture's protocol using Mut Express II Fast Mutagenesis Kit V2 (Vazyme, C214).

Primers for mutagenesis

No	Primer name	Sequence (5' to 3')
1	4TPR(327-1046)-fOGT fwd	CACAGCAGACTCTCTGAATAACCTAGCCAATAT
2	4TPR(327-1046)-fOGT rev	TTCAGAGAGTCTGCTGTGCTGTCGGCCACGTTG
3	2TPR(395-1046)-fOGT fwd	ACAGCTGATGCCTACTCTAATATGGGAAACACT
4	2TPR(395-1046)-fOGT rev	AGAGTAGGCATCAGCTGTGCTGTCGGCCACGTT
5	0TPR(463-1046)-fOGT fwd	ACAGCACACCTGATGCTTATTGTAACTTGGCTC
6	0TPR(463-1046)-fOGT rev	AAGCATCAGGTGTGCTGTCGGCCACGTTGCCCA
7	HTN-BRD4 Δ1051-1224 rev fwd	CACCACAAGGAGCAGTTCCGCCGCGCCGCTCGG
8	HTN-BRD4 Δ1051-1224 rev	GAACTGCTCCTTGTGGTGCCGGGGTGAATGGTG
9	HTN-BRD4 S1051A/ S1055A fwd	GgcggacccctacgcaACCGGTCACCTCCGCGAA
10	HTN-BRD4 S1051A/ S1055A rev	TtgcgtaggggtccgcCTTGTGGTGCCGGGGTGA
11	HTN-BRD4 1201A/1204A/1211A fwd	ccctagtgcagaagcatccggccACCCCCTCCTCCACAGCC

12	HTN-BRD4 1201A/1204A/1211A rev	atgcttctgcactagggcggcccaggcGCCCATGTTCTTGATTTTCAGG
13	HTN-CK2α S347A fwd	TCgccagcGCCAATATGATGTCAGGGATTTCTT
14	HTN-CK2α S347A rev	TCATATTGGCgctggcGACGGGCGTACTGCCCCC
15	HTN-CK2α S347A/S348A fwd	TCgccgccGCCAATATGATGTCAGGGATTTCTT
16	HTN-CK2α S347A/S348A rev	TCATATTGGCggcggcGACGGGCGTACTGCCCCC

All primers were synthesized by TechDragon Limited. The open reading frame of mutant plasmids were sequenced by BGI TECH SOLUTIONS (BEIJING LIUHE) CO., LIMITED. before conducting experiments.

Pulse-chase assay

1 X 10^5 HEK293T cells were seeded in each well of 12 well plate. After cells attached, transfection reagents and plasmids were prepared and added in DMEM. After 24 h transfection, media were replaced by fresh warm complete media with DMSO or compounds. After certain time of OGTACs treatment, media in wells were replaced by fresh warm media with 5 μM rhodamine ligand. The cells were incubated in 37 °C for 15 mins. Then, media were aspirated and 100 μL IP lysis buffer (Thermo Scientific 87788) supplemented with 20 μM OGA inhibitor (Thiamet G, Bidepharm BD571819) and 100X protease inhibitor (MedChemExpress, HY-K0010) (referred as complete IP lysis buffer in the later paragraph) was added to each well. The lysate was incubated on ice for 20 min, spun down at 14,000 RPM at 4°C for 15 mins, and the supernatant were collected for bicinchoninic acid (BCA) assay and normalized to a final 2 mg/mL. The lysates were denatured by SDS-loading buffer (Biorad #1610747) and 10 μL of each sample was loaded in SDS-PAGE. After running, in gel fluorescence at rhodamine channel (545/575 nm) was analysed by Bio-Rad ChemiDoc MP Imaging System. After image, the gel was transferred to PVDF membrane to check equal loading using anti-GAPDH antibody.

CETSA

HEK293T cells (2.5 X 10^6 cells) were seeded in 10 cm culture dish overnight for attachment. On the next day, cells were transfected with HTN-BRD4:fOGT=1:0.05 plasmid for 24 hours until ~90% confluence. After treatment with 5 μM compounds or DMSO, cells were collected and washed with PBS. Subsequently, cells were re-suspended in 500 μL PBS and equally divided into 50 μL aliquots. The tubes were subjected to heat challenge at 37-53°C for 5 mins, followed by cooling on ice. Cells were lysed by three repeated freeze-thaw cycles with liquid nitrogen and water bath. Lastly, cells were centrifuged at 14000 RPM for 15 mins and supernatants were collected for Western Blot analysis.

Immunoprecipitation

For each IP reaction, 2.2 X 10^6 HEK293T cells were seed in 10 cm dish. After cells attached, transfection reagents and plasmids were prepared and added in DMEM. After 24 h transfection, media were replaced by fresh warm complete media with DMSO or probes. For washout study, after 4 h treatment, media with DMSO or OGTAC-4 (500 nM) were replaced by fresh media with DMSO or AP1867 (50 μM) for further incubation. After certain time of OGTACs treatment, cells were collected by cold PBS and lysed by 200 μL complete IP lysis buffer. The lysate was incubated on ice for 20 min, spun down at 14,000 RPM at 4°C for 15 mins, and the supernatant were collected for bicinchoninic acid (BCA) assay and normalized to a final 2 mg/mL. For input, 20 μL of diluted sample was reacted with equal volume of 10 μM TMR ligand in IP lysis buffer and rotate gently at RT for 15 mins. The remaining samples were subjected to protein A/G magnetic beads (MedChemExpress, HY-K0202), which pre-binding with protein target protein antibody. For samples for LC-MS/MS or HTN-BRD4 mutant constructs, Halo-Trap Magnetic Agarose (Proteintech, otma) was used to incubate with protein lysates. After gentle rotation at 4 °C for at least 16 h, the beads were washed with IP lysis buffer and boiled in 40 μL 2X SDS-loading buffer (Biorad #1610747) for 5 mins to elute proteins from beads. Eluted samples were directly subjected into WB analysis. To get stronger signal of RL2, we used anti-mouse-HRP (Cell signaling, #7076) as secondary antibody for RL2 and anti-rabbit-DyLight 488 (Invitrogen, #35552) or IRDye® 680RD Goat anti-Rabbit (LI-COR, 926-68071) as secondary antibody for total target proteins. To clearly illustrate the overlay between the signal of RL2 and the total target proteins, we displayed the signal of RL2 and the corresponding total proteins in two colours on the same blot using Bio-Rad Image Lab software 6.1.0. Specifically, for **Figure 2**, **Figure 4C&D**, **Figure S1**, **Figure S3**, **Figure S10, Figure S13B**, **Figure S17B**, IRDye® 680RD Goat anti-Rabbit was used for target proteins and displayed in red, while the corresponding RL2 signal was displayed in green; for other remaining blots, anti-rabbit-DyLight 488 was used for target proteins and displayed in green, while the corresponding RL2 signal was displayed in red.

Chemoenzymatic labelling and mass shift assay

For each chemoenzymatic labelling reaction, 2 X 10^5 HEK293T cells were seed to each well of 6 well plate. After cells attached, transfection reagents and plasmids were prepared and added in DMEM. After 24 h transfection, media were replaced by fresh warm complete media with DMSO or probes. After certain time of OGTACs treatment, cells were collected by cold PBS and lysed by 200 μL RIPA lysis buffer (Thermo Scientific 89901) supplemented with 20 μM OGA inhibitor (Thaimet G, Bidepharm BD571819), 100X protease inhibitor (MedChemExpress, HY-K0010) and 1 μL/mL BeyoZonase (Beyotime, D7121). The supernatant of lysate was then quantified by bicinchoninic acid (BCA) assay and normalized to 2 mg/mL with 1 % SDS in buffer. Then, lysate was reduced by DTT (20 mM) at 58 °C for 1 h and alkylated by IAA (90 mM) for 40 mins in dark. For 100 μL lysate, added in sequence with vortex: 400 μL MeOH, 100 μL $CHCl_3$, 300 μL H_2O. The protein pellet was then washed with MeOH 500 μL and redissolved in 1% SDS GalT buffer. The following reagents were add in sequence: Protein lysate: 40 μL; H_2O: 49 μL; Label buffer: 80 μL; $MnCl_2$: 11 μL; UDP; GalNAz: 10 μL; Protease inhibitor: 2 μL; Gal-T Y289L: 5 μL; Final volume: 200 μL. The reaction was incubated at 4°C for 20 h with gentle rotation. The proteins were then precipitated, washed by MeOH and redissolved in 1 % SDS GalT buffer, and reacted with DBCO-PEG5k (final concentration 1 mM) for 5 mins at 95 °C. The protein were

then precipitated, washed by MeOH and redissolved in 1 X SDS-loading buffer for western blot analysis.

Western blotting and Antibody

In general, cells (from 12 well plate) were lysed by 80 μl RIPA lysis buffer (Thermo Scientific 89901) supplemented with 20 μM OGA inhibitor (Thaimet G, Bidepharm BD571819) and 100X protease inhibitor (MedChemExpress, HY-K0010), incubated on ice for 20 min, spun down at 14,000 RPM at 4°C for 15 mins, and the supernatant were collected for bicinchoninic acid (BCA) assay and normalizedeto a final 2 mg/mL concentration. About 30 μg of protein samples were loaded for sodium dodecyl sulfatepolyacrylamide gel electrophoresis (SDS-PADE) and blotted with indicated antibodies. Antibodies used in this study are as follow:

Antibody	Brand	Dilution (application)
RL2	Abcam (ab2739)	1:1000 (WB)
Anti-BRD4	Cell Signalling Technology (CST) (13440S)	1:2000 (WB)<br>1:100 (IP)
Anti-BRD4 (for mutant)	Abclonal (A12677)	1:1000 (WB)
Anti-OGT	CST (D1D8Q)	1:1000 (WB)
Anti-CK2α	CST (2656)	1:1000 (WB)
Anti-EZH2	CST (5246)	1:2000 (WB)<br>1:100 (IP)
Anti-CK2α	Proteintech (10992-1-AP)	1:50 (IP)
Anti-GAPDH	Santa Cruz (sc-47724)	1:3000 (WB)
Anti-HaloTag	Promega (G9211)	1:1000 (WB)
Goat anti-Mouse-HRP	CST (7076)	1: 6000 (WB)
Goat anti-rabbit-HRP	CST (7074)	1: 6000 (WB)
Goat anti-rabbit-DyLight 488	Invitrogen (35552)	1: 6000 (WB)
IRDye® 680RD Goat anti-Rabbit IgG (H + L)	LI-COR (926-68071)	1: 20000 (WB)
Normal rabbit IgG	CST (2729)	1:100 (IP)

Mass Spectrometry

For MS sample preparation, cell pellets were treated and collected same as the method in immunoprecipitation. HaloTrap Magnetic Agarose (proteintech, otma) was separated as 25 μL aliquot for each reaction. The beads were washed three times with 500 μL IP lysis buffer, followed by complete removal of supernatant. Then, cell lysates were added to each tubes and incubated at 4 °C for 4 h with gentle rotation. After incubation, beads were intensively washed with IP lysis buffer and eluted in 2 X SDS-loading buffer heated at 95 °C for 5 mins. The samples were submitted to SDS-PAGE and stained with Coomassie brilliant blue solution, and the bands around 250 kDa were cut out, washed three times by ddH_2O, destained in 100 μL destaining buffer for 20 mins at 25 °C and this process was repeated once. The gel was then washed by 100% ACN for 15 mins and dried. Then tris(2-carboxyethyl) phosphine (TCEP) was added to reduce protein for 30 mins at 25 °C, followed by addition of iodoacetamide (IAA) solution for 30 mins reaction at 25 °C in dark. The supernatant was discarded

and wash by 100% ACN, freeze dried. The sample was then digested by Trypsin solution for 20 h at 37 °C. After centrifugation, supernatant was collected and digested peptides were extracted by the following steps. Extraction solution was added and incubated for 20 mins at 25 °C; this step was repeated once and 100% ACN was added to extracted again. All extracted fractions were combined and freeze dried and desalt by C18 columns. The elutes from columns were freeze dried and injected to MS.

Mass spectrometry acquisition procedures

LC-MS/MS data was collected by timsTOF Pro2 mass spectrometer coupled with a nanoElute UPLC system (Bruker Daltonics, Bremen, Germany). The peptides were dissolved in phase A (0.1% formic acid in water). 100 ng of peptides were analysed by C18 column (Aurora Series, 75 µm × 25 cm, Ionopticks, Victoria, Australia) with the gradient set as: 0-48 min, 4-18% solvent B (0.1% formic acid in ACN); 48-55 min, 18-35% B; 55-57 min, 35-95% B; 57-60 min, 95% B with flow rate at 300 nL/min. Peptides were analysed using DDA mode by LC-MS/MS. The parameters set as follows: scan range (m/z) = 300-1500; tims scan range or 1/K0 range (V·s/cm2) = 0.75-1.35; MS1 resolution = 60,000; Target Intensity = 100000; Intensity Threshold=2500; number of PASEF MS/MS scans=6; Total cycle time=1.16s; charge range = 2–5; Isolation Width: 2 m/z (when <800m/z), 3 m/z (when > 800 m/z);

Mass spectrometry data analysis

The raw data were processed using PEAKS Studio (version 11, Bioinformatics Solutions Inc., Waterloo, Canada) against Homo_sapiens proteome in UniProt/SwissProt human (Homo sapiens) protein database plus our construct (HTN-BRD4). The parameters set as follows: Precursor Mass Error Tolerance: 20.00ppm, Fragment Mass Error Tolerance: 0.05Da, Enzyme: Trypsin, Max Missed Cleavage: 2, Digest Mode: Specific, Peptide Length Range: 5 - 45, Max Variable PTM per Peptide: 3, Fixed Modifications: Carbamidomethylation (+57.02), Variable Modifications: HexNAcylation (ST) (+203.08), Oxidation (M) (+15.99).Database: Homo_sapiens proteome (20387 proteins) plus our construct (HTN-BRD4), Taxonomy: all species, Searched Entries: 1, Deep Learning Boost: Yes, Report Filter: higher than 1% false discovery rate (FDR) < 1% and protein unique peptides >= 2; the O-GlcNAc modification specific- peptide-to-spectrum match (PSM) using characteristic peak with > 1% ion intensity. The O-GlcNAcylation modification occupancy was calculated by detected modified peptides intensity divided by detected total peptide intensity for each sample.

Chemical synthesis

NMR spectra were acquired on Bruker 400 & 500 NMR spectrometer, running at 400 MHz for ^{1}H and Bruker 500 NMR spectrometer at 126 MHz for ^{13}C respectively. ^{1}H

NMR spectra were recorded at 400 MHz in $CDCl_3$, using residual $CHCl_3$ as the internal standard. ^{13}C NMR spectra were recorded at 126 MHz in $CDCl_3$ using residual $CHCl_3$ as the internal standard. Reactions were monitored by thin layer chromatography and the products were purified using preparative thin layer flash chromatography (ALUGRAM Xtra, 818333). Mass spectrometry was performed on Agilent LC-MS/MS system consisted of two Agilent 1290 series pumps and auto-sampler, coupled with 6430 triple quadrupole mass spectrometer equipped with and ESI source (Agilent Technologies, Inc., Santa Clara, CA, USA). Unless otherwise noted, analytical grade solvents and commercially available reagents were used without further purification. Unless otherwise noted, chemical starting materials are purchased from Bide pharm without further purification.

(R)-1-(3-(2-((2-(2-((6-chlorohexyl)oxy)ethoxy)ethyl)amino)-2-oxoethoxy)phenyl)-3-(3,4-dimethoxyphenyl)propyl (S)-1-((S)-2-(3,4,5-trimethoxyphenyl)butanoyl)piperidine-2-carboxylate (OGTAC-1) and analogues

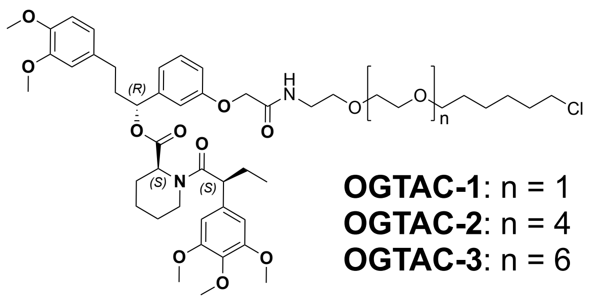

OGTAC-1: n = 1
OGTAC-2: n = 4
OGTAC-3: n = 6

General method: Synthesis of OGTAC-1/2/3 were adapted from literature.[2] Take OGTAC-1 as an example, the 2-[3-[(1R)-3-(3,4-dimethoxyphenyl)-1-[(2S)-1-[(2S)-2-(3,4,5-trimethoxyphenyl)butanoyl]piperidine-2-carbonyl]oxy-propyl]phenoxy]acetic acid (55.2 mg, 0.079 mmol) (**AP1867**, synthesized according to literature[3,4]) was dissolved in DMF (1.5 mL), 1-[Bis(dimethylamino)methylene]-1H-1,2,3-triazolo[4,5-b]pyridinium 3-oxide hexafluorophosphate (HATU) (35 mg, 0.1 mmol), and DIPEA (50 µL, 0.35 mmol) were added and stirred for 30 minutes. NH_2-PEG_2-C6-Cl (20 mg, 0.1 mmol) was added. The reaction mixture was stirred overnight. The reaction mixture was extracted with ethyl acetate and water, purified on preparative TLC (ALUGRAM Xtra, 818333), and evaporated under vacuum to give product as clear oil (26 mg, 35 %). Product confirmed by ESI–MS m/z: 899.6 $[M+H]^+$, 921.5 $[M+Na]^+$, 937.6 $[M+K]^+$,; and ^1H-NMR according to literature.[2] Other probes are synthesised using same reagents just changing NH_2-PEG_2-C6-Cl to NH_2-PEG_5-C6-Cl, NH_2-PEG_7-C6-Cl, and the products were confirmed by ESI–MS and ^1H-NMR according to literature.[2].

(R)-3-(3,4-dimethoxyphenyl)-1-(3-(2-((2-(2-(hexyloxy)ethoxy)ethyl)amino)-2-oxoethoxy)phenyl)propyl (S)-1-((S)-2-(3,4,5-trimethoxyphenyl)butanoyl)piperidine-2-carboxylate

(OGTAC-1H)

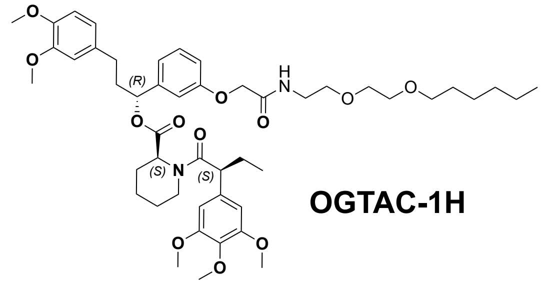

OGTAC-1H

It was synthesized as the general procedures above. AP1867 (56 mg, 0.08 mmol) was dissolved in DMF (2 mL), 1-[Bis(dimethylamino)methylene]-1H-1,2,3-triazolo[4,5-b]pyridinium 3-oxide hexafluorophosphate (HATU) (35 mg, 0.1 mmol), and DIPEA (50 μL, 0.35 mmol) were added and stirred for 30 minutes. NH_2-PEG_2-C6 (34 mg, 0.15 mmol) (BCLP-38, Xi'an Confluore Biological Technology Co., Ltd.) was added. The reaction mixture was stirred overnight. The reaction mixture was extracted with ethyl acetate and water, purified on preparative TLC (ALUGRAM Xtra, 818333), and evaporated under vacuum to give product as clear oil (28 mg, 40 %). Product confirmed by ESI–MS m/z: 865.7.6 $[M+H]^+$, 887.7 $[M+Na]^+$. ^{1}H-NMR: δ 7.20–7.14 (m, 1H), 6.80 – 6.74 (m, 3H), 6.69–6.61 (m, 3H), 6.40 (d, J = 2.0 Hz, 2H), 5.62 (dd, J = 8.2, 5.5 Hz, 1H), 5.47–5.43 (m, 1H), 4.49 (m, 2H), 3.88–3.80 (m, 11H), 3.78 (s, 3H), 3.68 (s, 6H), 3.50-3.62 (m, 12H), 3.43 (td, J = 6.7, 1.6 Hz, 2H), 2.84-2.73 (m, 1H), 2.63-2.40 (m, 4H), 2.17-2.01 (m, 3H), 1.76–1.50 (m, 5H), 0.90 (m, 8H); ^{13}C NMR: δ 172.77, 170.67, 168.34, 157.39, 153.28, 148.96, 147.44, 142.38, 136.71, 135.40, 133.44, 129.91, 120.27, 119.84, 114.09, 112.85, 111.76, 111.35, 105.05, 104.64, 75.77, 71.67, 70.46, 70.06, 69.80, 67.40, 60.87, 56.38, 56.06, 56.01, 55.93, 52.16, 50.88, 43.58, 38.95, 38.37, 31.77, 31.37, 29.80, 29.67, 28.44, 26.90, 25.85, 25.43, 22.71, 21.03, 14.15, 12.66.

4-((2-(2-((6-chlorohexyl)oxy)ethoxy)ethyl)carbamoyl)-2-(6-(dimethylamino)-3-(dimethyliminio)-3H-xanthen-9-yl)benzoate (rhodamine ligand)

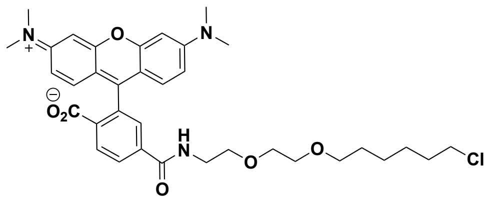

rhodamine ligand

This probe was synthesised following the method described in literature.[5] 5(6)-TAMRA NHS ester (14 mg, 0.027 mmol) was dissolved in DMF (2 mL) and ten equivalents diisopropyethylamine (DIPEA) (24 µL) was added to the resultant solution. Then NH_2-PEG_2-C6-Cl (10 mg, 0.039 mmol) was added to reaction. The reaction was protected from light and reacted for 8 h. Subsequently, the product was dissolved in water and freeze dried, followed by reconstitution in MeOH. The product was purified by PTLC to get final product as dark red powder (15 mg, 86 %). Product confirmed by ESI–MS m/z: 646 $[M+H]^+$.

(R)-1-(3-(2-((6-((tert-butoxycarbonyl)amino)hexyl)amino)-2-oxoethoxy)phenyl)-3-(3,4-dimethoxyphenyl)propyl (S)-1-((S)-2-(3,4,5-trimethoxyphenyl)butanoyl)piperidine-2-carboxylate (AP1867-C6-NHBoc)

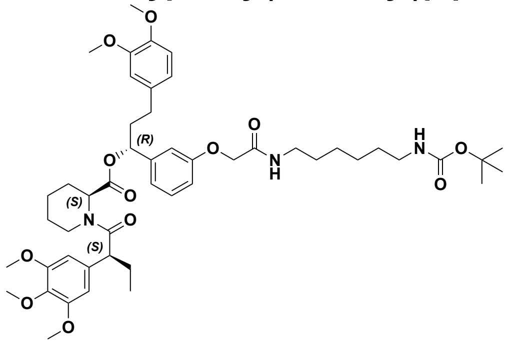

To a round bottom flask (RBF), AP1867 (57.8 mg, 0.08 mmol) was added and dissolved in 2 mL dry DMF. Then DIPEA (70 µL) and HATU (45.6 mg, 0.12 mmol) were added to the solution and stirred for 15 mins. N-Boc-1,6-diaminohexane (25.3 mg, 0.1 mmol) was then added and stirred for another 16 h. The reaction was then partitioned in EtOAc and H_2O, extracted with EtOAc and the dried over Na_2SO_4. The crude product was purified by PTLC (EtOAc: Hexane= 2 : 1) to have clear oil (33 mg, 51 %). ESI–MS m/z: 691 $[M+H]^+$(without Boc). ^{1}H-NMR: δ 7.21 – 7.14 (m, 1H), 6.83 – 6.77 (m, 3H), 6.72– 6.63 (m, 3H), 6.43 (d, J = 2.0 Hz, 2H), 5.67 (dd, J = 8.2, 5.5 Hz, 1H), 5.49 – 5.45 (m, 1H), 4.50 (m, 2H), 3.88 – 3.81 (m, 10H), 3.78 (s, 3H), 3.68 (s, 5H), 3.61 (t, J = 6.7 Hz, 1H), 3.40-3.31 (m, 2H), 3.15-3.06 (td, 2H), 2.86-2.76 (t, 1H), 2.65-2.47 (m, 2H), 2.37-2.28 (m, 1H), 2.15-1.98 (m, 4H), 1.79 – 1.25 (m, 5 H), 1.60 (s, 6H), 1.45 (s, 9H), 0.93 (t, 3H); ^{13}C-NMR: δ 172.86, 170.73, 168.32, 157.43, 153.30, 148.99, 147.78, 142.47, 136.73, 135.40,133.45, 129.95, 120.31, 119.96, 113.65, 113.13, 111.80, 111.38, 105.07, 75.73, 60.90, 56.43, 56.04, 55.97, 52.20, 50.91, 43.60, 40.70, 39.10,38.36, 31.42, 30.06, 29.83, 29.59, 28.45, 26.92, 26.55, 26.42, 25.44, 21.02, 12.86, 12.69.

(*R*)-1-(3-(2-((6-(2-((*S*)-4-(4-chlorophenyl)-2,3,9-trimethyl-6*H*-thieno[3,2-*f*][1,2,4]triazolo[4,3-*a*][1,4]diazepin-6-yl)acetamido)hexyl)amino)-2-oxoethoxy)phenyl)-3-(3,4-dimethoxyphenyl)propyl (*S*)-1-((*S*)-2-(3,4,5-trimethoxyphenyl)butanoyl)piperidine-2-carboxylate (OGTAC-4)

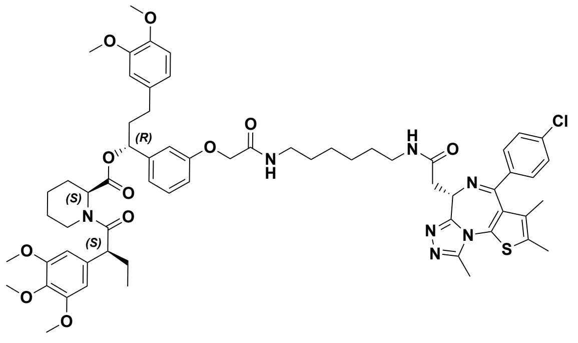

To a RBF, JQ-1 (carboxylic acid, MedChemExpress, HY-78695) (13.2 mg, 0.03 mmol) was added and dissolved in 1 mL dry DMF. Then DIPEA (26 µL) and HATU (17 mg) were added to the solution and stirred for 15 mins. **AP1867-C6-NHBoc** (20 mg, 0.03 mmol) was then dissolved in 1 mL dry DMF and added. The reaction was stirred for another 16 h. The reaction was then partitioned in EtOAc and H_2O, extracted with EtOAc and the dried over Na_2SO_4. The crude product was purified by PTLC (MeOH: DCM= 1 : 30) to have white solid (28 mg, 79 %). ESI–MS m/z: 1175.5 $[M+H]^+$, 1196.5 $[M+Na]^+$. ^{1}H-NMR: ^{1}H-NMR: δ 7.39 (d, J = 8.4 Hz, 2H), 7.31 (d, J = 8.4 Hz, 2H), 7.21 – 7.14 (m, 1H), 6.83 – 6.77 (m, 3H), 6.72– 6.63 (m, 3H), 6.43 (d, J = 2.0 Hz, 2H), 5.67 (dd, J = 8.2, 5.5 Hz, 1H), 5.49 – 5.45 (m, 1H), 4.63 (t, 1H), 4.50 (m, 2H), 3.88 – 3.81 (m, 10H), 3.78 (s, 3H), 3.68 (s, 5H), 3.61 (t, J = 6.7 Hz, 1H), 3.39-3.30 (m, 2H), 2.86-2.76 (t, 1H), 2.68 (s, 3H), 2.65-2.47 (m, 2H), 2.42 (s, 3H), 2.37-2.28 (m, 1H), 2.15-1.98 (m, 5H), 1.79 – 1.25 (m, 6H), 1.68 (s, 3H), 1.81-1.31 (m, 8H), 0.93 (t, 3H); ^{13}C-NMR: δ 172.86, 170.71, 168.19, 164.65, 157.44, 153.27, 150.15, 148.95, 147.43, 142.39, 136.68, 135.41,133.47, 132.03, 131.75, 131.30, 130.64, 130.20, 129.90, 128.92, 120.29, 119.86, 113.69, 113.11, 111.79, 111.70, 105.05, 76.9, 70.64, 60.99, 56.39, 56.06, 55.96, 54.41, 52.16, 51.31, 43.57, 38.95, 38.90,38.31, 32.39, 31.38, 29.55, 28.48, 28.45, 26.88, 25.41, 21.00, 13.26, 12.82.

Reference

(1) Levine, Z. G.; Potter, S. C.; Joiner, C. M.; Fei, G. Q.; Nabet, B.; Sonnett, M.; Zachara, N. E.; Gray, N. S.; Paulo, J. A.; Walker, S. Mammalian Cell Proliferation Requires Noncatalytic Functions of O-GlcNAc Transferase. *Proc. Natl. Acad. Sci. U. S. A.* **2021**, *118* (4), 1–11. https://doi.org/10.1073/PNAS.2016778118.

(2) Chen, P.; Hu, Z.; An, E.; Okeke, I.; Zheng, S.; Luo, X.; Gong, A.; Jaime-figueroa, S.; Crews, C. M. Modulation of Phosphoprotein Activity by Phosphorylation Targeting Chimeras (PhosTACs). **2021**. https://doi.org/10.1021/acschembio.1c00693.

(3) Amara, J. F.; Clackson, T.; Rivera, V. M.; Guo, T.; Keenan, T.; Natesan, S.; Pollock, R.; Yang, W.; Courage, N. L.; Holt, D. A.; Gilman, M. A Versatile Synthetic Dimerizer for the Regulation of Protein-Protein Interactions. *Proc.*

Natl. Acad. Sci. U. S. A. **1997**, *94* (20), 10618–10623. https://doi.org/10.1073/pnas.94.20.10618.
(4) Clackson, T.; Yang, W.; Rozamus, L. W.; Hatada, M.; Amara, J. F.; Rollins, C. T.; Stevenson, L. F.; Magari, S. R.; Wood, S. A.; Courage, N. L.; Lu, X.; Cerasoli, F.; Gilman, M.; Holt, D. A. Redesigning an FKBP-Ligand Interface to Generate Chemical Dimerizers with Novel Specificity. *Proc. Natl. Acad. Sci. U. S. A.* **1998**, *95* (18), 10437–10442. https://doi.org/10.1073/pnas.95.18.10437.
(5) Liße, D.; Wilkens, V.; You, C.; Busch, K.; Piehler, J. Selective Targeting of Fluorescent Nanoparticles to Proteins inside Live Cells. *Angew. Chemie - Int. Ed.* **2011**, *50* (40), 9352–9355. https://doi.org/10.1002/anie.201101499.
